# PSMD12-Mediated M1 Ubiquitination of Influenza A Virus at K102 Regulates Viral Replication

**DOI:** 10.1128/jvi.00786-22

**Published:** 2022-07-21

**Authors:** Xianfeng Hui, Lei Cao, Ting Xu, Lianzhong Zhao, Kun Huang, Zhong Zou, Peilei Ren, Haiying Mao, Ying Yang, Shuo Gao, Xiaomei Sun, Xian Lin, Meilin Jin

**Affiliations:** a State Key Laboratory of Agricultural Microbiology, Huazhong Agricultural University, Wuhan, China; b College of Animal Medicine, Huazhong Agricultural University, Wuhan, China; c Key Laboratory of development of veterinary diagnostic products, Ministry of Agriculture, Wuhan, Hubei, People’s Republic of China; d The Cooperative Innovation Center for Sustainable Pig Production, Wuhan, China; Lerner Research Institute, Cleveland Clinic

**Keywords:** influenza A virus, M1 protein, PSMD12, K63-linked ubiquitination, K102 site, replication

## Abstract

The M1 of influenza A virus (IAV) is important for the virus life cycle, especially for the assembly and budding of viruses, which is a multistep process that requires host factors. Identifying novel host proteins that interact with M1 and understanding their functions in IAV replication are of great interest in antiviral drug development. In this study, we identified 19 host proteins in DF1 cells suspected to interact with the M1 protein of an H5N6 virus through immunoprecipitation (IP)/mass spectrometry. Among them, PSMD12, a 26S proteasome regulatory subunit, was shown to interact with influenza M1, acting as a positive host factor in IAV replication in avian and human cells. The data showed that PSMD12 promoted K63-linked ubiquitination of M1 at the K102 site. H5N6 and PR8 with an M1-K102 site mutant displayed a significantly weaker replication ability than the wild-type viruses. Mechanistically, PSMD12 promoted M1-M2 virus-like particle (VLP) release, and an M1-K102 mutation disrupted the formation of supernatant M1-M2 VLPs. An H5N6 M1-K102 site mutation or knockdown PSMD12 disrupted the budding release of the virus in chicken embryo fibroblast (CEF) cells, which was confirmed by transmission electron microscopy. Further study confirmed that M1-K102 site mutation significantly affected the virulence of H5N6 and PR8 viruses in mice. In conclusion, we report the novel host factor PSMD12 which affects the replication of influenza virus by mediating K63-linked ubiquitination of M1 at K102. These findings provide novel insight into the interactions between IAV and host cells, while suggesting an important target for anti-influenza virus drug research.

**IMPORTANCE** M1 is proposed to play multiple biologically important roles in the life cycle of IAV, which relies largely on host factors. This study is the first one to identify that PSMD12 interacts with M1, mediates K63-linked ubiquitination of M1 at the K102 site, and thus positively regulates influenza virus proliferation. PSMD12 promoted M1-M2 VLP egress, and an M1-K102 mutation affected the M1-M2 VLP formation. Furthermore, we demonstrate the importance of this site to the morphology and budding of influenza viruses by obtaining mutant viruses, and the M1 ubiquitination regulator PSMD12 has a similar function to the M1 K102 mutation in regulating virus release and virus morphology. Additionally, we confirm the reduced virulence of H5N6 and PR8 (H1N1) viruses carrying the M1-K102 site mutation in mice. These findings provide novel insights into IAV interactions with host cells and suggest a valid and highly conserved candidate target for antiviral drug development.

## INTRODUCTION

Annually, influenza A virus (IAV) is responsible for approximately 290,000 to 650,000 deaths worldwide (1). The influenza virus relies not only on viral proteins but also on host cell proteins and their associated mechanisms to complete the viral life cycle (2). Hence, a better understanding of how viral components interact with and utilize host molecules is critical to elucidate the mechanisms of pathogenesis and provide valuable targets for antiviral therapy.

Interactions between host proteins and those of the influenza virus are an important mechanism in the regulation of viral replication and pathogenicity (3). Replication of the influenza virus is dependent strongly on host cells; the virus completes its own replication cycle by “hijacking” the host cell’s biological processes and utilizing its energy, lipids, RNA, and proteins. Interacting with host proteins, the influenza virus can alter or utilize the host’s normal physiological processes to create an environment that facilitates its own replication and then complete replication (4). The M1 matrix protein, a 252-residue structural protein in the influenza virus that oligomerizes into an endoskeleton-like coat beneath the viral lipid bilayer, is one of the most abundant and most highly conserved proteins of IAV. M1 plays a critical role during virus budding and assembly (5–7). Despite its functional importance, few studies have explored which host proteins are involved in regulating the function of M1 in the life cycle of influenza viruses and their specific molecular mechanisms.

In recent years, a novel reassortant highly pathogenic avian influenza virus (HPAIV), H5N6, has replaced H5N1 as one of the dominant IAV subtypes circulating in waterfowl and causing human infections in China (8–10). Frequent outbreaks of H5N6 virus have caused great economic losses and pose a severe threat to human health. Birds, particularly waterfowl, play a central role in the preservation and transmission of the influenza virus (11). Therefore, the goal of the current study was to investigate proteins in chicken embryo fibroblast DF1 cells that interact with the M1 protein of H5N6 virus in an effort to identify a host protein implicated in the life cycle of the influenza virus and determine its mechanism of action. The study findings present a previously unknown host protein required for influenza viral replication both in chicken DF1 and human A549 cells and suggest a highly conserved amino acid site on M1 for antiviral drug development.

## RESULTS

### Confirmation of 8 candidate genes involved in the proliferation of influenza A virus of H5N6.

**FIG 1:**
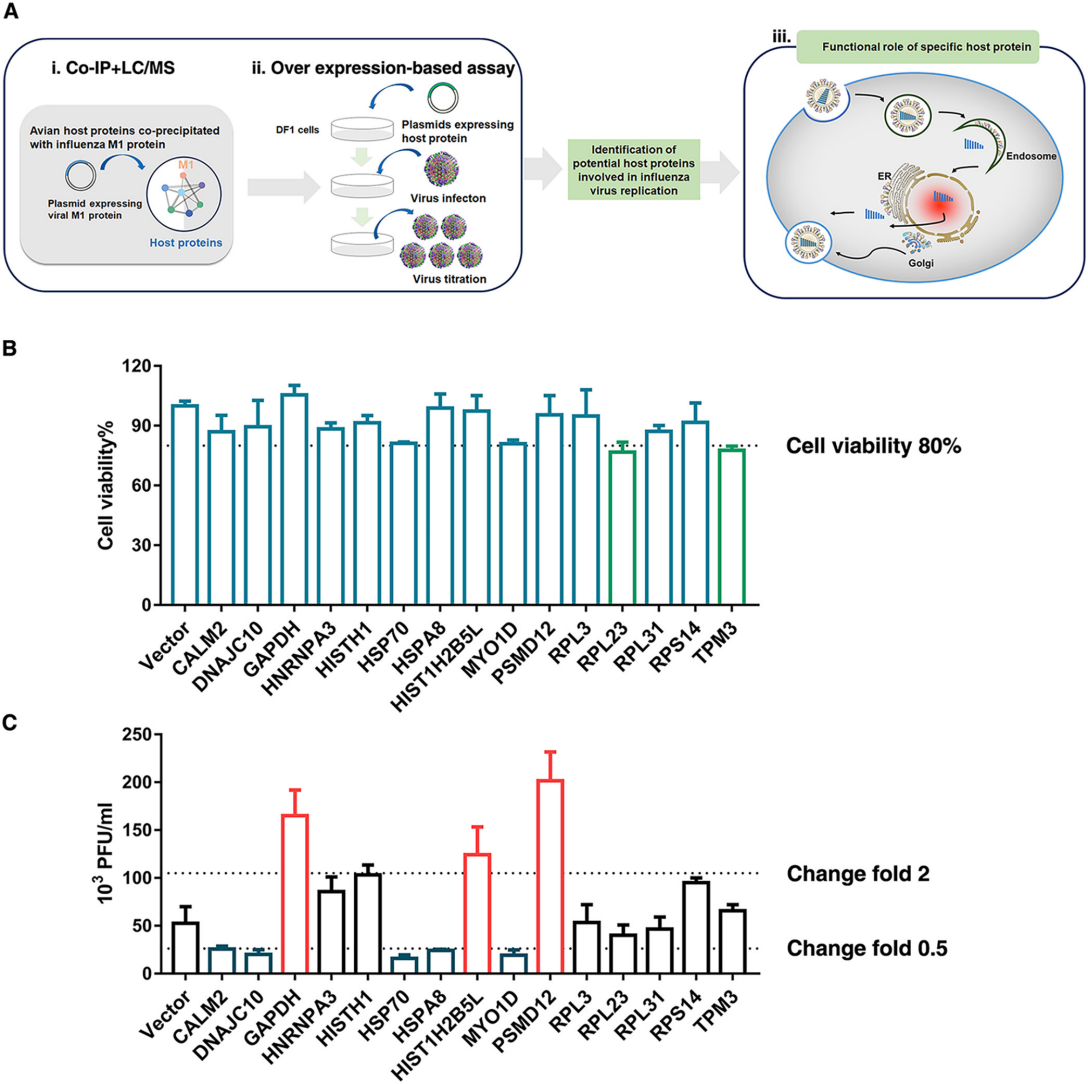
PSMD12 was identified to positively regulate the proliferation of the H5N6 influenza virus. (A) Schematic diagram of the identification of host proteins that coprecipitated with influenza A viral protein M1. (i) Mass spectrometry was used to identify host proteins that coimmunoprecipitated with HA-tagged influenza viral protein M1. (ii) To identify host factors that affected viral replication, cells were transfected with plasmids to overexpress each of the candidate host genes and then infected with influenza virus. Virus titers and cell viability were then determined. (iii) Based on the screening results, the molecular mechanisms of the candidate host proteins were further explored. (B) DF1 cells were transfected with the indicated plasmids, infected with H5N6 virus at an MOI of 0.05, and the supernatant was collected for viral plaque measurement. (C) Viability of DF1 cells transfected with the indicated plasmids.

We first attempted to determine the influenza M1-host protein interactions in chicken embryo fibroblast (CEF) DF1 cells that were implicated in H5N6 virus replication (schematic diagram in Fig. 1A). A hemagglutinin (HA)-tagged M1 protein of H5N6 was expressed in DF1 cells, immunoprecipitated with an anti-HA antibody, and analyzed by silver staining and immunoblotting (see Fig. S1A in the supplemental material). Mass spectrometry analysis identified 19 host proteins that coprecipitated with the viral M1 protein (Fig. S1B). From the 19 host factors (see Table S1 in the supplemental material) identified by immunoprecipitation/mass spectrometry (IP/MS), 15 were successfully cloned into pCAGGS vectors. DF1 cells were then transfected with plasmids overexpressing each of the candidate host genes. Before the above overexpression verification experiment, the efficiency of the Lipofectamine 2000 reagent on DF1 cell transfection was evaluated by transfection of the GFP-expression plasmid. The results indicated that nearly 50% of the cells could effectively express GFP-M1 (see Fig. S2A and B in the supplemental material), supporting further screening experiments. After 24 h posttransfection, cell viability was measured using the CCK-8 assay (Fig. 1B), and cells were infected with the H5N6 virus at a multiplicity of infection (MOI) of 0.05. At 24 hours postinfection (hpi), the culture supernatant was harvested for the plaque assay. Finally, we validated 3 positively regulated genes (greater than 2-fold increase in number of viral plaques) and 5 negatively regulated genes (less than 0.5-fold increase in number of viral plaques) (Fig. 1C). Among them, PSMD12 had the strongest positive regulatory effect on viral proliferation.

### Knockdown of PSMD12 inhibits H5N6 proliferation in avian DF1 cells and human A549 cells.

**FIG 2:**
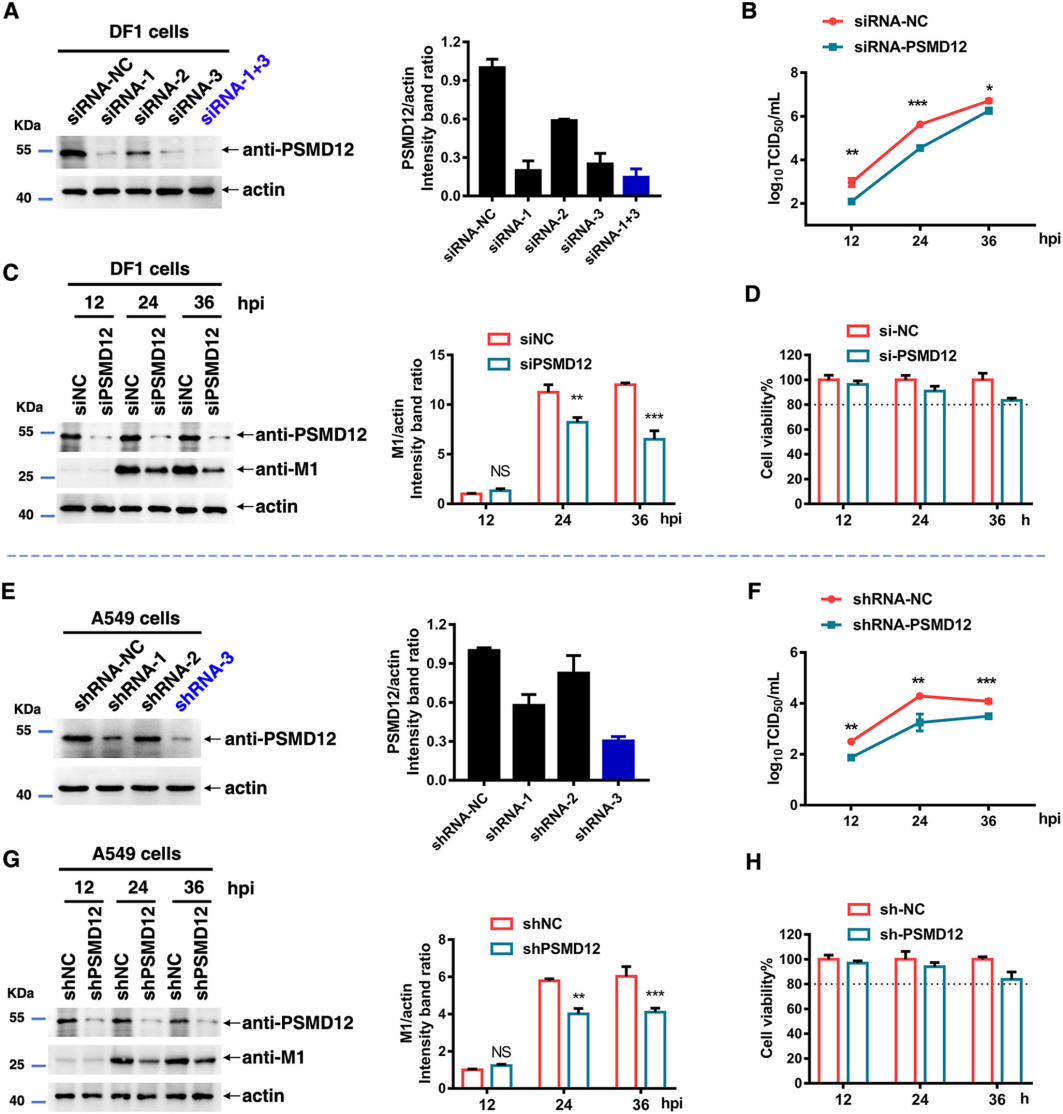
Knockdown of PSMD12 inhibits H5N6 virus replication. (A) DF1 cells were transfected with siRNA-PSMD12 or siRNA-negative control (NC), and cells were harvested and analyzed via Western blotting (WB). (B, C) DF1 cells were infected with H5N6 virus 24 h after transfection with siRNAs. Supernatant was collected at different time points for TCID_50_ measurement (B), and the cells were harvested and analyzed via WB (C). (D) Cell viability was determined at the indicated time points. (E) The effect of knockdown by different shRNAs in A549 cells was analyzed via WB. (F, G) PSMD12-knockdown A549 cells were infected with H5N6 virus, and the supernatant was collected at different time points for TCID_50_ measurement (F); cells were harvested and analyzed via WB (G). (H) Cell viability was determined at the indicated time points. Error bars, mean ± SD of three experiments. All comparisons were tested by two-tailed Student's *t* test; *, *P* < 0.05; **, *P* < 0.01; ***, *P* < 0.001.

To further verify the regulatory effect of PSMD12 on influenza virus, we designed three small interfering RNA (siRNAs) to target PSMD12 in avian DF1 cells (Fig. 2A) (the sequences of siRNA and short hairpin RNA [shRNA] are listed in Table S2 in the supplemental material). The knockdown efficiency of siRNA or shRNA was detected by Western blot. The combination of siRNA#1 and #3 (siRNA-1+3) had the ability to knockdown PSMD12 by more than 80% on DF1 cells (Fig. 2A). To evaluate PSMD12 knockdown effectiveness on the proliferation of H5N6 virus, DF1 cells were infected with H5N6 at 24 h post-siRNA transfection. Supernatants were collected at different time points for 50% tissue culture infective dose (TCID_50_) measurement, and cells were harvested for Western blot analysis. The data showed that the impaired expression of PSMD12 significantly decreased viral titers after 24 and 36 h compared with that of control CEF cell (siNC) transfection (Fig. 2B). Besides, there is a modest decrease of M1 levels after siRNA silencing of PSMD12 suggesting that PSMD12 has a modest regulation of M1 steady-state levels (Fig. 2C). In addition, to rule out the possibility that silencing PSMD12 may cause cytotoxicity in DF-1 cells, we carried out a cell viability assay, which showed that knockdown of PSMD12 had no obvious influence on cell viability (Fig. 2D).

It is well known that avian influenza viruses have the ability to infect humans. To evaluate the effect of knockdown PSMD12 on the proliferation of avian influenza viruses of H5N6 in human cells, three shRNAs were designed to target PSMD12 in human A549 cells. As shown in Fig. 2E, the efficiency of knockdown PSMD12 of shRNA-3 on A549 cells was about 70%. To evaluate PSMD12 knockdown effectiveness on the proliferation of H5N6 virus in human cells, we further challenged A549-shNC and A549-shPSMD12 cells with H5N6 virus for 12, 24, and 36 h. Similarly, the results showed that the impaired expression of PSMD12 significantly decreased viral titer (Fig. 2F) and M1 protein (Fig. 2G). In addition, the result of the cell viability assay ruled out the possibility that silencing PSMD12 might lead to a significant decrease of A549 cells viability (Fig. 2H). These results suggest that both avian and human PSMD12 have a positive regulatory effect on the AIV of H5N6.

### PSMD12 interacts with M1.

**FIG 3:**
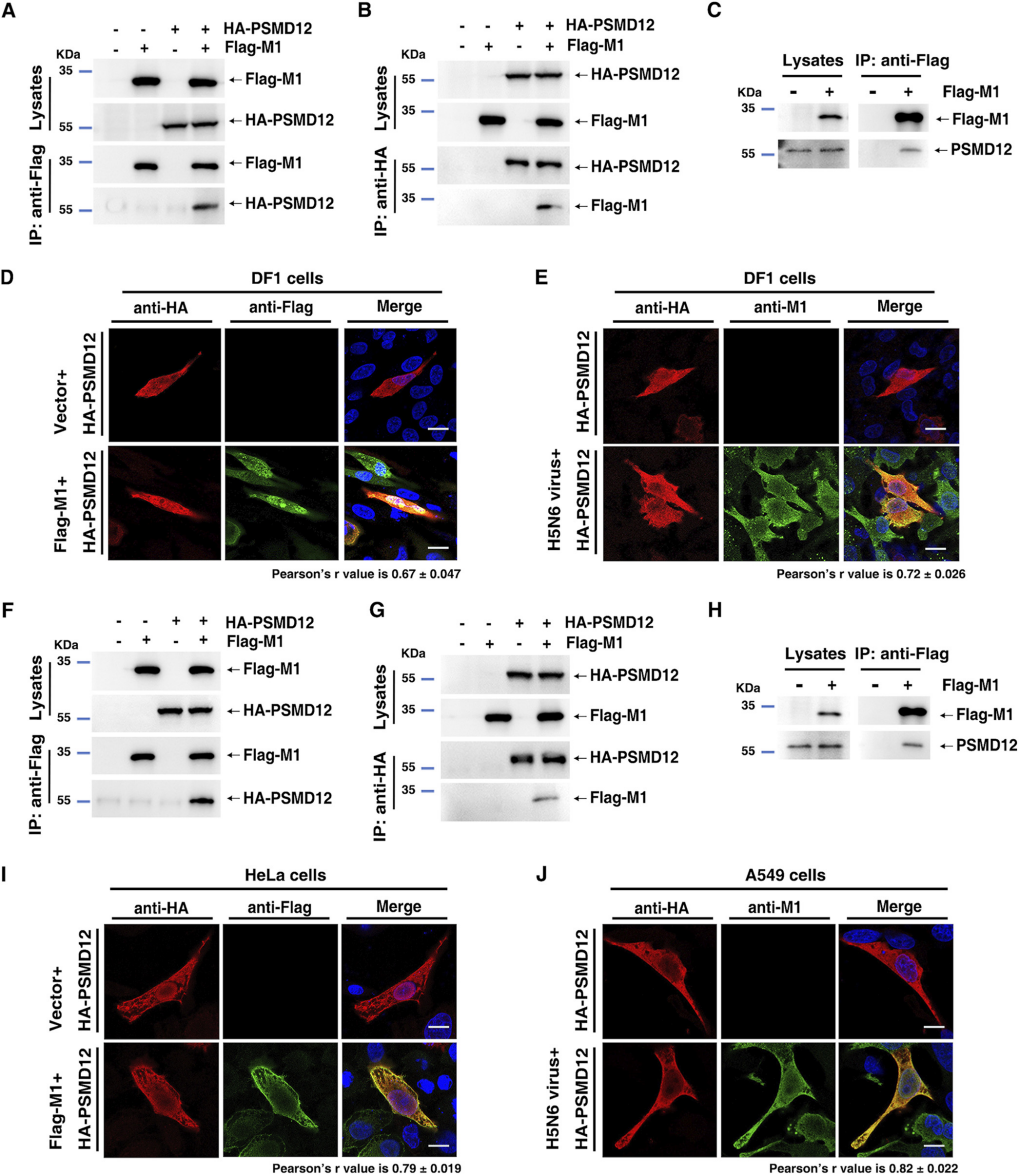
PSMD12 interacted with M1. (A) DF1 cells were transfected with plasmids encoding HA-PSMD12 and Flag-M1. The lysates were subjected to anti-Flag IP and analyzed via Western blotting (WB). (B) DF1 cells were transfected with plasmids encoding HA-PSMD12 and Flag-M1. The lysates were subjected to anti-HA IP and analyzed via WB. (C) DF1 cells were transfected with a plasmid encoding Flag-M1, and the lysate was subjected to IP and analyzed for endogenous PSMD12 via WB. (D, E) DF1 cells were transfected with plasmids encoding Flag-M1 (H5N6 virus) and HA-PSMD12 (avian source) and analyzed for colocalization of Flag-M1 and HA-PSMD12. Scale bar, 10 μm (D). DF1 cells were transfected with HA-PSMD12 plasmid, infected with H5N6 virus, and analyzed for colocalization of HA-PSMD12 and M1 of H5N6 virus. Scale bar, 10 μm (E). (F) HEK293T cells were transfected with plasmids encoding HA-PSMD12 (human source) and Flag-M1 (H5N6 virus). The lysates were subjected to anti-Flag IP and analyzed via WB. (G) HEK293T cells were transfected with plasmids encoding HA-PSMD12 and Flag-M1. The lysates were subjected to anti-HA IP and analyzed via WB. (H) HEK293T cells were transfected with a plasmid encoding Flag-M1, and the lysate was subjected to IP and analyzed for endogenous PSMD12 via WB. (I, J) HeLa cells were transfected with plasmids encoding Flag-M1 (H5N6 virus) and HA-PSMD12 (human source) and analyzed for colocalization of Flag-M1 and HA-PSMD12. Scale bar, 10 μm (I). A549 cells were transfected with an HA-PSMD12 plasmid, infected with H5N6 virus, and analyzed for colocalization of HA-PSMD12 and M1 of H5N6 virus. Scale bar, 10 μm (J). Pearson correlation coefficients were determined for molecular colocalization in confocal microscopy images using Coloc 2 of Fiji (regions of interest were chosen within 3 to 5 cells each group, *n* = 12). Fig. S3 contains scanned complete images of Western blots reference to this figure.

To elucidate the mechanism underlying the regulation of influenza virus replication by PSMD12, we first performed co-IP assays to investigate whether PSMD12 interacted with M1. As expected, Flag-M1 physically interacted with HA-PSMD12 (Fig. 3A and B) and endogenous PSMD12 coimmunoprecipitated with Flag-M1 in DF1 cells (Fig. 3C). Furthermore, the confocal microscopy results revealed that HA-PSMD12 colocated with Flag-M1 in DF1 cells (Fig. 3D), with Pearson’s *r* value of 0.67 ± 0.047, which measures how well two fluorescent foci coincide (12). To observe the interaction between PSMD12 and M1 in the viral life cycle, DF1 cells transfected with the HA-PSMD12 plasmid were infected with an H5N6 virus. The confocal microscopy results showed colocalization of HA-PSMD12 and M1 of the H5N6 virus, with a Pearson’s *r* value of 0.72 ± 0.026 (Fig. 3E). Further studies focused on human PSMD12 have also demonstrated the above conclusion; a co-IP experiment showed that Flag-M1 physically interacted with HA-PSMD12 (human source) (Fig. 3F and G) and endogenous PSMD12 coimmunoprecipitated with Flag-M1 in HEK293T cells (Fig. 3H). The confocal microscopy results presented that HA-PSMD12 (human source) colocated with Flag-M1 in HeLa cells (Pearson’s *r* value is 0.79 ± 0.019) (Fig. 3I) and colocated with M1 in the viral life cycle in A549 cells (Pearson’s *r* value is 0.82 ± 0.022) (Fig. 3J). Together, these results provided evidence supporting that influenza virus M1 interacted with PSMD12.

### PSMD12 mediates K63-linked M1 ubiquitination.

**FIG 4:**
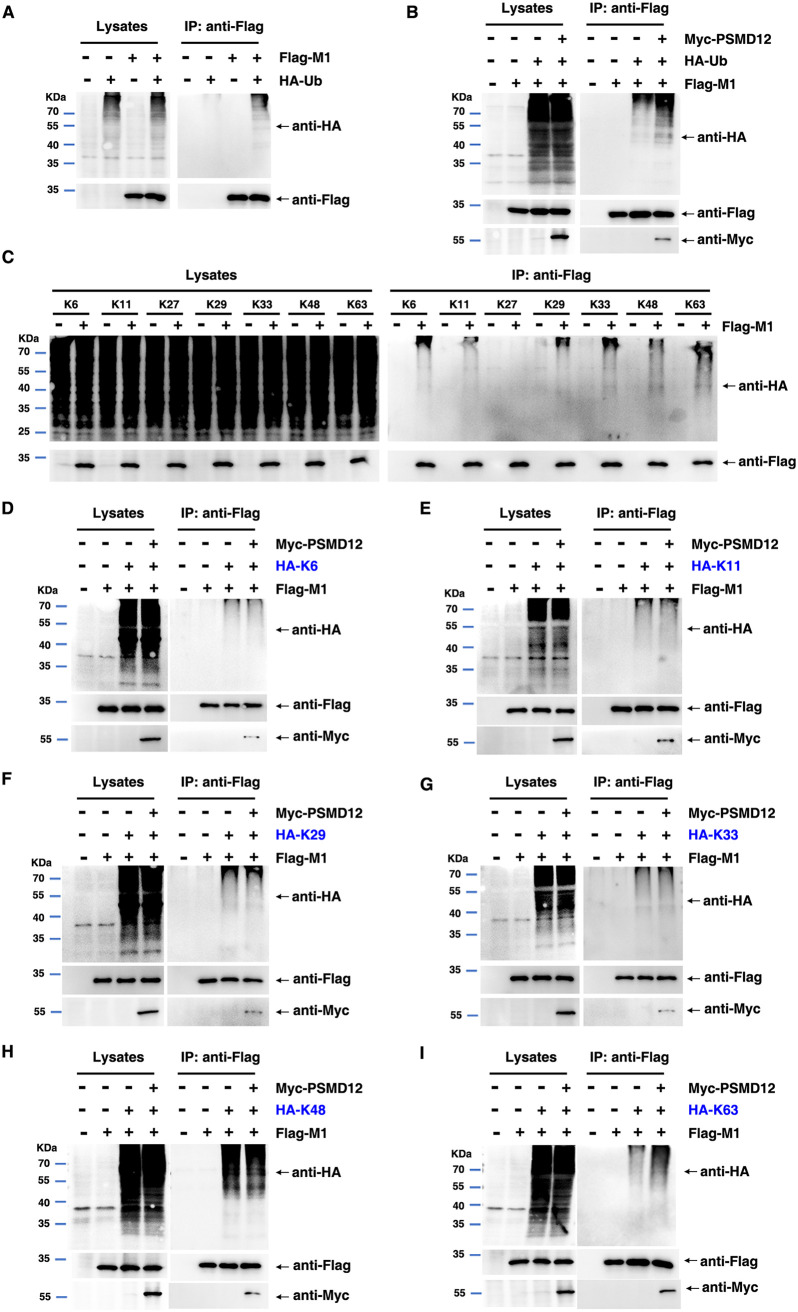
Host protein PSMD12 triggers K63-linked M1 ubiquitination. (A) HEK293T cells were transfected with the indicated plasmid combinations to measure ubiquitination of M1. (B) HEK293T cells overexpressing PSMD12 were transfected with the indicated plasmid combinations to measure ubiquitination of 1. (C) HEK293T cells overexpressing PSMD12 were transfected with the indicated plasmid combinations to measure K6/K11/K27/K29/K33/K48/K63-linked ubiquitination of M1. (D) HEK293T cells overexpressing PSMD12 were transfected with the indicated plasmid combinations to measure K6-linked ubiquitination of M1. (E) HEK293T cells overexpressing PSMD12 were transfected with the indicated plasmid combinations to measure K11-linked ubiquitination of M1. (F) HEK293T cells overexpressing PSMD12 were transfected with the indicated plasmid combinations to measure K29-linked ubiquitination of M1. (G) HEK293T cells overexpressing PSMD12 were transfected with the indicated plasmid combinations to measure K33-linked ubiquitination of M1. (H) HEK293T cells overexpressing PSMD12 were transfected with the indicated plasmid combinations to measure K48-linked ubiquitination of M1. (I) HEK293T cells overexpressing PSMD12 were transfected with the indicated plasmid combinations to measure K63-linked ubiquitination of M1.

Generally, the 26S proteasome is associated closely with the modification of cell proteins, including ubiquitination (13). To investigate whether PSMD12 (26S proteasome regulatory subunit) regulated the ubiquitination of M1, HEK293T cells were transfected with plasmids encoding Flag-M1 and HA-Ub, along with Myc-tagged PSMD12. The results clearly demonstrated that M1 can be ubiquitinated (Fig. 4A). Interestingly, ubiquitination of M1 was enhanced by PSMD12 overexpression (Fig. 4B). There are seven lysine residues within ubiquitin allowing for site-specific ubiquitination to occur at K6, K11, K27, K29, K33, K48, or K63 (14). To explore the type of ubiquitin chain linkage in M1, we cotransfected seven types of ubiquitin plasmids with empty vector or Flag-M1 for ubiquitination detection, and the results showed that the ubiquitination signal of Flag-M1 was obviously enhanced after cotransfection with K6, K11, K29, K33, K48, and K63 but not with K27 (Fig. 4C). This result suggests that M1 can be ubiquitinated by K6, K11, K29, K33, K48, and K63. To determine whether PSMD12-mediated M1 ubiquitination occurred via K6, K11, K29, K33, K48, or K63, we employed HA-tagged K6, K11, K29, K33, K48, and K63 to perform ubiquitination assays. The results indicated that overexpression of PSMD12 obviously increased K63-linked (Fig. 4I), but not K6-, K11-, K29-, K33-, or K48-linked ubiquitination of M1 (Fig. 4D to H).

### K102 is important for K63-linked ubiquitination of M1 mediated by PSMD12 and an M1-PSMD12 interaction.

**FIG 5:**
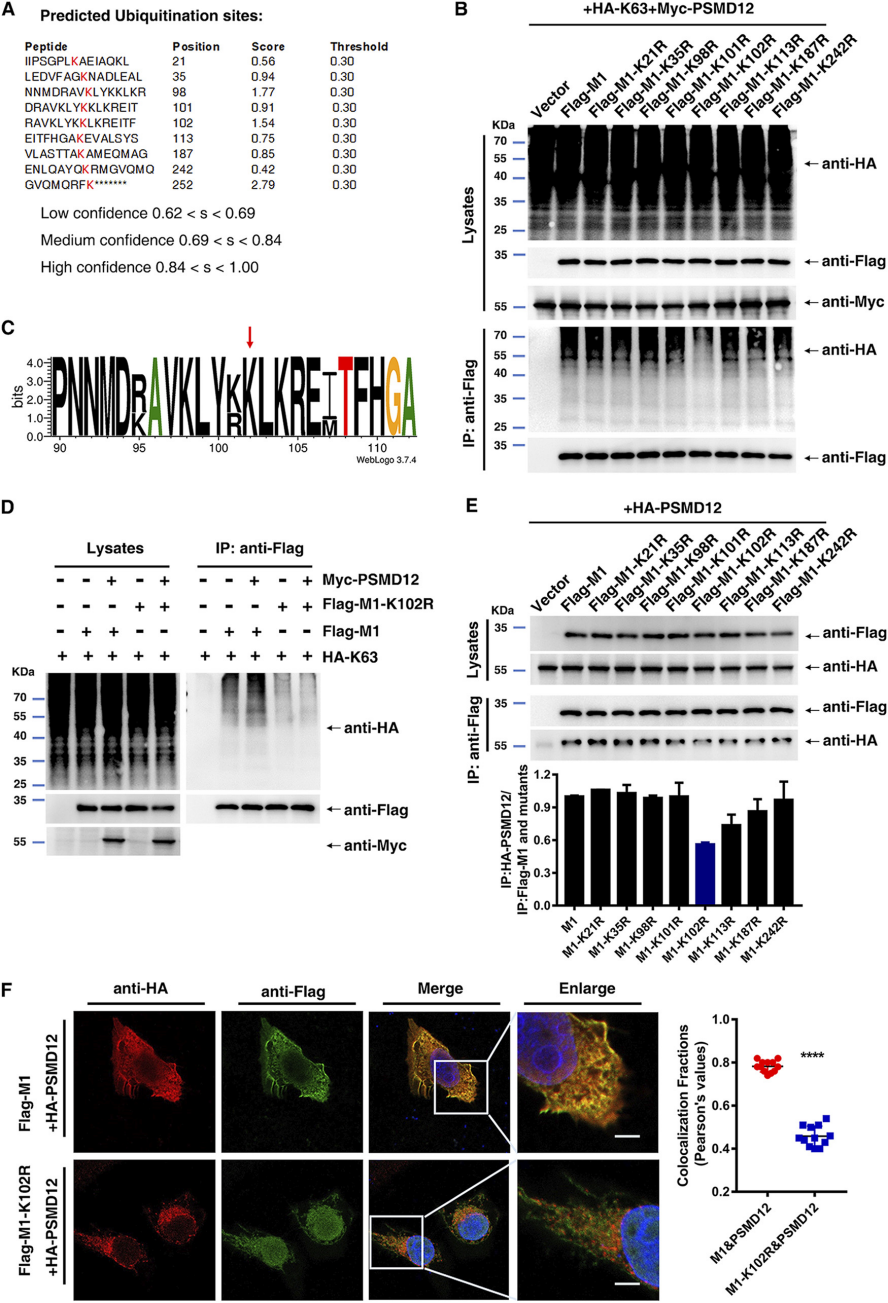
The K102 site is critical for K63-linked ubiquitination of M1 and K102R impairs the interaction between M1 and PSMD12. (A) The ubiquitination sites of M1 were predicted by BDM-PUB and UBPRED. (B) HEK293T cells were transfected with the indicated plasmid combinations to measure K63-linked ubiquitination of M1. (C) WebLogo output for amino acid sequences of M1 from H1N1, H2N2, H3N2, H5N1, H5N6, H7N9, and H9N2 influenza viruses. (D) HEK293T cells overexpressing Myc-PSMD12 and HA-K63 were transfected with the indicated plasmid combinations to measure the ubiquitination of PR8 Flag-M1/M1-K102R. (E) Interaction between HA-PSMD12 and wild-type and mutant M1. (F) HeLa cells were transfected with plasmids encoding Flag-M1/M1-K102R and HA-PSMD12, followed by analysis for colocalization of Flag-M1/M1-K102R and HA-PSMD12. Scale bar, 10 μm. Pearson correlation coefficients were determined for molecular colocalization in confocal microscopy images using Coloc 2 of Fiji (regions of interest were chosen within 3 to 5 cells each group, *n* = 12).

Bioinformatics methods were employed to predict the possible ubiquitination sites of M1 from H5N6. Based on the intersection predicted by BDM-PUB (http://bdmpub.biocuckoo.org/results.php) and UBPRED (http://www.ubpred.org/) software (Fig. 5A), eight lysine residues (K21, K35, K98, K101, K102, K113, K187, and K242) were selected to construct M1 mutants (K to R, respectively). HEK293T cells were transfected with plasmids encoding wild-type (WT) and H5N6-M1 mutants K to R, together with HA-K63 and Myc-PSMD12. The results of a subsequent ubiquitination assay showed that PSMD12-mediated K63-linked ubiquitination of M1 was obviously reduced after mutation at K102 of M1 (Fig. 5B). The PROSITE sequence logo for M1 of influenza virus (15), shown in Fig. 5C, indicated that the K102 site is conserved. We additionally explored the effect of the M1-K102 site mutation on K63-linked ubiquitination of the PR8 virus. The results of a subsequent ubiquitination assay indicated that the K102 site mutation significantly reduced the K63-linked ubiquitination of M1 and blocked K63-linked ubiquitination of M1 mediated by PSMD12 (Fig. 5D). Ubiquitination is an important posttranslational modification that regulates function, localization, and interaction (16, 17). To verify whether the M1-K102 site is associated with an M1-PSMD12 interaction, co-IP assays were performed. The data showed that the K102 site mutation weakened the interaction between M1 and PSMD12 most obviously among the eight M1 mutants (K21R, K35R, K98R, K101R, K102R, K113R, K187R, and K242R). Furthermore, immunofluorescence experiments were performed after HeLa cells were cotransfected with plasmids encoding Flag-M1/Flag-M1-K102R and HA-PSMD12. The results revealed that the colocalization signal of Flag-M1-K102R and HA-PSMD12 was obviously weaker than that of Flag-M1 and HA-PSMD12 (Fig. 5F). From these data, we calculated the Pearson’s coefficient of correlation to measure if the two fluorescent foci coincide. Results showed that the colocalization fractions of M1 and PSMD12 were significantly higher than that of M1-K102R and PSMD12 (Fig. 5F). Together, these findings suggested that the K102 not only is an important site for K63-linked ubiquitination of M1 mediated by PSMD12 but also contributes to the interaction between M1 and PSMD12.

### M1-K102 site mutation significantly reduced viral proliferation, and this site is associated with the proliferation of H5N6 regulated by PSMD12.

**FIG 6:**
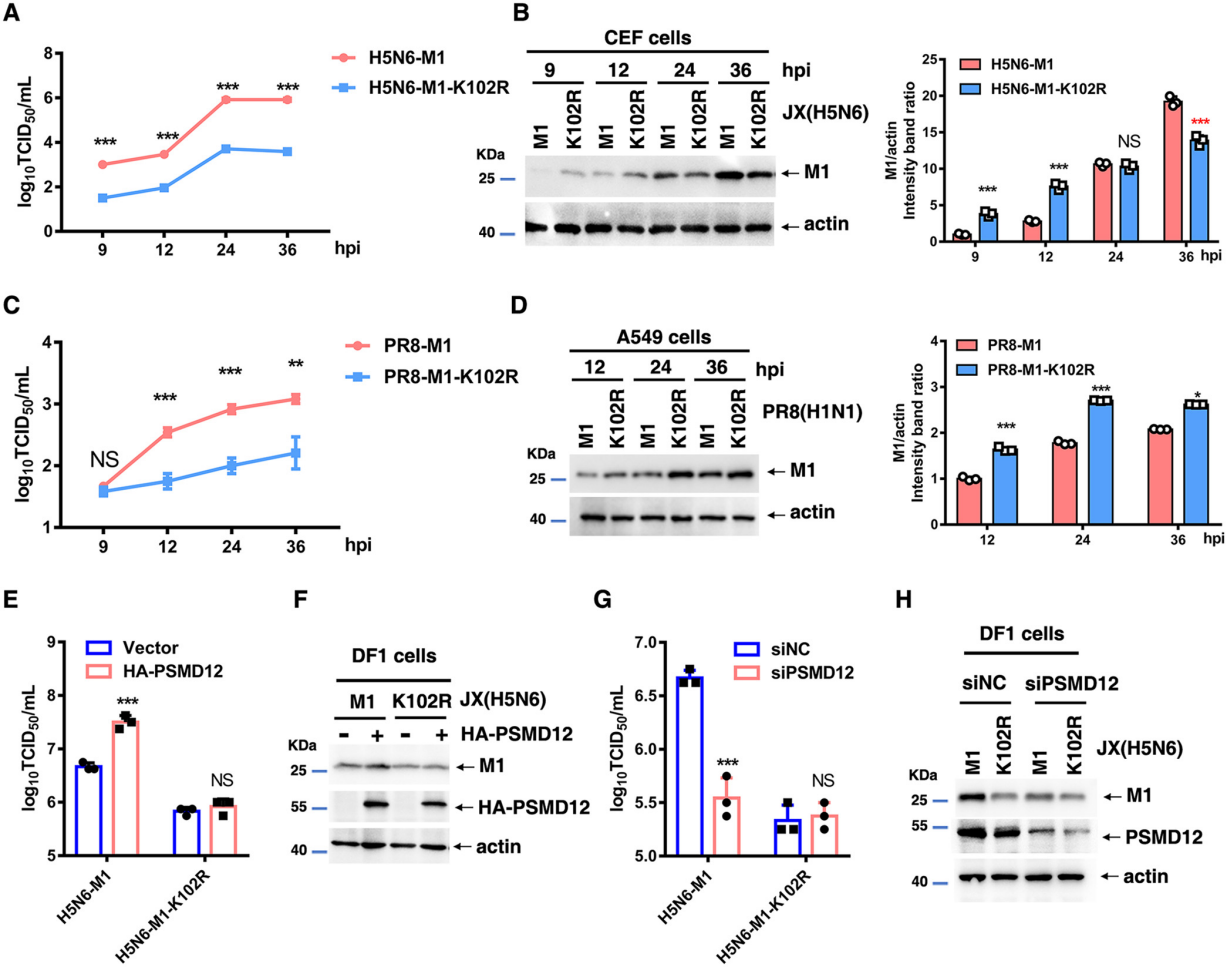
Mutation at the M1 K102 site alters viral replication in cells and PSMD12 fails to regulate M1 K102 mutant H5N6 proliferation. (A, B) CEF cells were infected with H5N6-M1 or H5N6-M1-K102R virus, the supernatant was collected at different time points for TCID_50_ measurement, and the cell lysates were analyzed by WB. (C, D) A549 cells were infected with PR8-M1 or PR8-M1-K102R virus, the supernatant was collected at different time points for TCID_50_ measurement, and the cell lysates were analyzed by WB. (E, F) DF1 cells were transfected with the indicated plasmids and infected with H5N6-M1 or H5N6-M1-K102R virus at an MOI of 0.05. The supernatant was collected at 24 h postinfection for TCID_50_ measurement (E), and cells were harvested and analyzed via WB (F). (G, H) DF1 cells were infected with H5N6-M1 or H5N6-M1-K102R virus at an MOI of 0.05 after transfection with siRNAs. The supernatant was collected at 24 h postinfection for TCID_50_ measurement (G), and cells were harvested and analyzed via WB (H). Error bars, mean ± SD of three experiments. All comparisons were tested by two-tailed Student’s *t* test; *, *P* < 0.05; **, *P* < 0.01; ***, *P* < 0.001.

To investigate the effect of an M1-K102 site mutation in the life cycle of the influenza virus, we generated mutated viruses with a single mutation at K102, named H5N6-M1-K102R and PR8-M1-K102R viruses (see Fig. S4A and B in the supplemental material). These mutations were further confirmed by sequencing analysis (Fig. S4C and D). In contrast to infection with the H5N6-M1 (H5N6-WT) virus, infection with the H5N6-M1-K102R virus resulted in significantly decreased viral yield in the cultural supernatant of chicken embryo fibroblast (Fig. 6A) and Madin-Darby canine kidney (MDCK) cells (see Fig. S5A in the supplemental material). Similarly, in contrast to infection with the PR8-M1 (PR8-WT) virus, infection with the PR8-M1-K102R virus resulted in significantly decreased viral yield in the cultural supernatant of A549 (Fig. 6C) and MDCK cells (Fig. S5C). Notably, although the viral yield was reduced in the supernatant of cells infected with H5N6-M1-K102 or PR8-M1-K102R virus, the M1 level was significantly increased in the cell lysate compared with that of cells infected with the respective WT viruses (H5N6-M1 or PR8-M1) (Fig. 6B and D, Fig. S5B and D). These results suggested that a mutation of M1 at K102 may block the release of the virus into the supernatant.

Furthermore, to assess the replication of WT and M1 mutant viruses in control and PSMD12-overexpressing or -knockdown cells. H5N6-M1 and H5N6-M1-K102R were used to infect control, PSMD12-overexpressed, or PSMD12-knocked down DF1 cells. The supernatant was collected at 24 h postinfection for TCID_50_ measurement, and cells were harvested and analyzed via Western blotting. Our results showed that PSMD12 overexpression promoted H5N6 virus proliferation (Fig. 6E and F), whereas PSMD12 knock down inhibited the proliferation of H5N6 on DF1 cells (Fig. 6G and H). However, neither overexpression nor knockdown of PSMD12 could significantly affect the proliferation of H5N6-M1-K102R on DF1 cells. Taken together, these findings provided further evidence that the mechanism of PSMD12 positively regulating the proliferation of H5N6 virus was related to the K102 site of M1.

### M1 K102 mutation or knockdown of PSMD12 restricts virus release.

**FIG 7:**
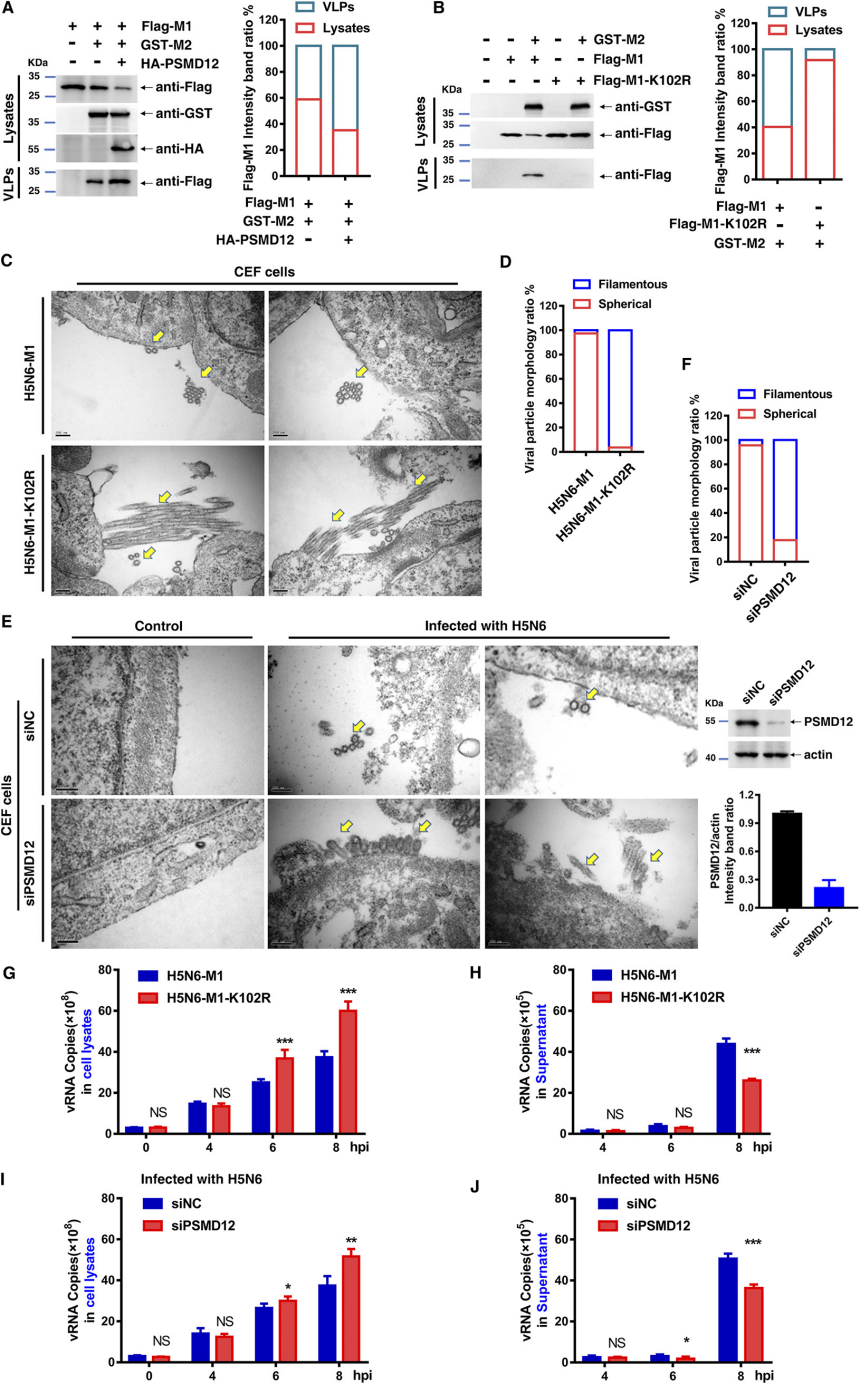
M1 K102 mutation or knockdown PSMD12 restricts virus release. (A, B) HEK293T cells were transfected with the indicated plasmid combinations to detect the influence on the egress of M1. (C) CEF cells were infected with H5N6-M1 or H5N6-M1-K102R virus at an MOI of 0.1 for 10 h followed by transmission electron microscopy. The yellow arrows indicate virus particles. Scale bar, 200 nm. (D) Quantification of virion morphology under 10 random fields. Virus particle length greater than 2-fold the virion diameter are defined as filamentous phenotype. (E) CEF cells (siNC and siPSMD12) were infected with H5N6 virus at an MOI of 0.1 for 10 h followed by transmission electron microscopy. The yellow arrows indicate virus particles. Scale bar, 200 nm. The efficiency of siRNA knockdown was evaluated by Western blotting. (F) Quantification of virion morphology under 10 random fields. Virus particles length greater than 2-fold the virion diameter are defined as filamentous phenotype. (G, H) DF1 cells were infected with H5N6-M1 or H5N6-M1-K102R virus (MOI of 5); the cell lysates (G) and supernatant (H) were collected at different time points, and vRNA of M1 was analyzed by qRT-PCR. (I, J) DF1 cells were infected with H5N6-M1 virus at an MOI of 5 after transfection with siRNAs. The cell lysates (I) and supernatant (J) were collected at different time points, and vRNA of M1 was analyzed by qRT-PCR. Error bars, mean ± SD of three experiments. All comparisons were done by two-tailed Student’s *t* test; *, *P* < 0.05; **, *P* < 0.01; ***, *P* < 0.001.

With evidence that mutant viruses H5N6-M1-K102R and PR8-M1-K102R were blocked from release in the culture supernatant, we sought to determine whether the M1-K102 site mutation or knockdown PSMD12 affected budding of the virus. The M1 of IAV is viewed generally as a key orchestrator in the release of influenza virions from the plasma membrane during infection (18). However, in the absence of other viral proteins, M1 by itself fails to form virus-like particles (VLPs). Notably, M2 effectively targets M1 to the plasma membrane and produces extracellular M1 VLPs (18). Therefore, we first investigated whether PSMD12 and its modified M1-K102 site affect M1-M2 VLP release before the virus budding experiment. As shown in Fig. 7A, Flag-M1 alone could not be released into the supernatant; however, M1 was detected in the supernatant after cotransfection with M2. Interestingly, overexpression of PSMD12 promoted the release of M1 VLPs in the supernatant, suggesting that PSMD12 promoted the formation of M1-M2 VLPs. Since PSMD12 mediates the ubiquitination of M1 at the K102 site and the M1 K102 site is involved in PSMD12-regulated viral proliferation, we speculate that the M1 K102 site mutation might affect M1-M2 VLP release. To test this conjecture, HEK293T cells were cotransfected with plasmids encoding Flag-M1 or Flag-M1-K102R and GST-M2. The supernatant and cells were collected for Western blotting, revealing that the release of Flag-M1-K102R in the supernatant was decreased compared with that of Flag-M1 (Fig. 7B). We further examined the budding of influenza viruses in CEF cells by transmission electron microscopy (TEM). As expected, virions on the surface of cells infected by the H5N6-M1-K102R virus remained attached to the plasma membrane as opposed to the completely released IAV particles after infection with the WT virus (Fig. 7C), which suggested that a virus budding defect phenotype was associated with the mutation at K102 of M1. In addition, the morphology of H5N6-M1-K102R was obviously different from that of H5N6-M1. Specifically, most of the mutant virus particles were filamentous and only a few were spherical (Fig. 7D). To confirm whether PSMD12 affects the budding of H5N6 virus, PSMD12 knockdown CEF cells obtained by using siRNA (siRNA usage is the same as on DF1 cells above, and the knockdown efficiency of siRNA is shown in Fig. 7E [the right]) was infected with H5N6-M1 to perform a transmission electron microscopy experiment. Results showed that knockdown PSMD12 also inhibited the budding and release of H5N6 on CEF cells (Fig. 7E), and H5N6-M1 appeared morphologically bacilliform or even filamentous on PSMD12 knocked down CEF cells (siPSMD12), while it was spherical on control CEF (siNC) cells (Fig. 7F). These results suggest that PSMD12 and M1 K102 have a similar influence on the regulation of H5N6 virus budding and morphology.

To further understand the requirements of the M1 K102 site or PSDM12 for virion release, single-cycle high MOI infection experiments were performed. The DF1 cells were infected with H5N6-M1 or H5N6-M1-K102R virus at an MOI of 5, and cell lysates and supernatants were collected at different time points within 8 h (single-cycle replication of IAV) after infection. The viral RNA (vRNA) was detected in cellular supernatants and cell lysates by reverse transcription-quantitative PCR (qRT-PCR). The results showed that there was no significant difference in vRNA of M1 in cell lysates at the initial stage of infection (0 hours postinfection [hpi] and 4 hpi), but the vRNA of M1 of H5N6-M1-K102R was significantly higher than that of H5N6-M1 at 6 hpi and 8 hpi (Fig. 7G). In parallel to this finding, the vRNA of both viruses (H5N6-M1 and H5N6-M1-K102R) in supernatant was significantly higher than that in the initial stage of infection at 8 hpi. Importantly, the vRNA of H5N6-M1-K102R was significantly lower than that of H5N6-M1 (Fig. 7H). Additionally, we performed PR8-M1 or PR8-M1-K102R single-cycle high MOI infection experiments on A549 cells (see Fig. S6A and B in the supplemental material). The results were consistent with those of DF1 cells infected with H5N6-M1 or H5N6-M1-K102R. In short, mutations at the M1 K102 site affect the virus released to supernatant. To further understand the requirements of host PSDM12 for virion release, PSMD12 was knocked down in DF1 cells using siRNA and infected with H5N6-M1. The results showed that the knock down of PSMD12 increased vRNA in cell lysates and decreased vRNA in the supernatant (Fig. 7I and J). To verify whether this phenomenon was applicable to PR8 influenza virus, we used PR8 influenza virus to infect A549 control or PSMD12 knockdown cells with shRNA (Fig. S6C and D). The results were consistent with those of DF1 cells infected with H5N6-M1, and knockdown of PSMD12 interfered with influenza A virus release to the supernatant. Together, these results suggest that PSMD12 and M1 K102 have similar trends of influence on the regulation of influenza A virus release.

It is well known that neuraminidase (NA) plays an important role in the life cycle of influenza virus by assisting mature influenza virus in detaching from host cells and infecting new cells (19). To evaluate whether the mutation at the M1 K102 site resulted in restricted budding release of the virus by affecting viral NA activity, we performed NA activity assays. The results showed that the NA activities of H5N6-M1-K102R and PR8-M1-K102R mutant viruses were not significantly different from those of the corresponding wild-type viruses (see Fig. S7A and B in the supplemental material).

### M1-K102R mutation reduces viral pathogenicity in mice.

Given that the single mutation at position K102 of M1 in both H5N6 and PR8 viruses restricted the release of M1 and virus, we next determined the pathogenicity of M1-K102R mutant viruses in C57/BL6 mice. Before this experiment, 50% median lethal dose (MLD50) values were determined for the two WT viruses in mice. As shown in Fig. S8A to D in the supplemental material, the MLD50 values of H5N6-M1 and PR8-M1 viruses were 1.50 × 10^6^ TCID_50_ and 3.75 × 10^5^ TCID_50_, respectively.

**FIG 8:**
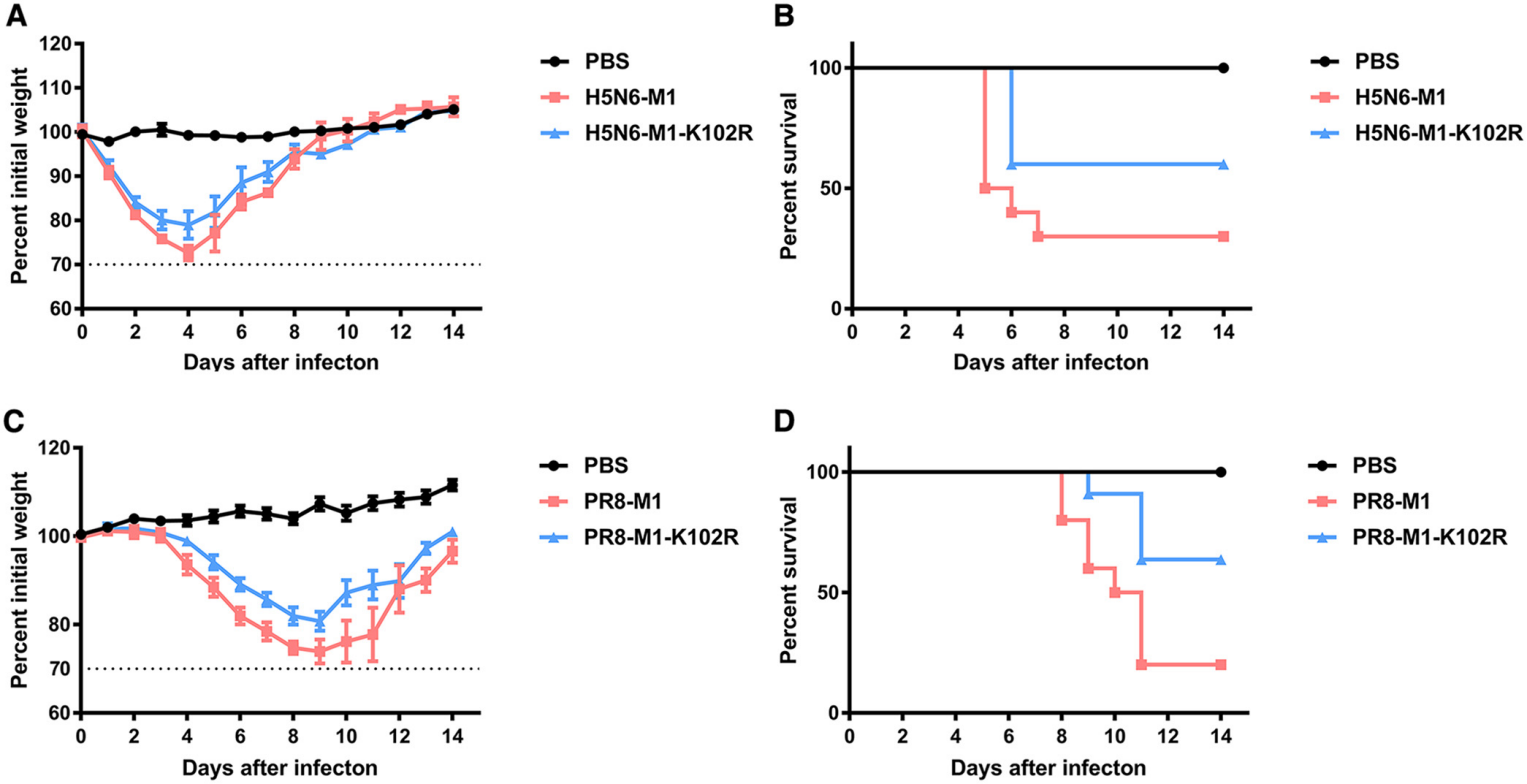
M1-K102 site mutation of influenza A virus affects its pathogenicity in mice. (A, B) Six-week-old C57/BL6 mice were infected with the same doses of H5N6-M1 and H5N6-M1-K102R viruses. Body weight (A) and survival (B) were monitored daily for 2 weeks (*n* = 10). (C, D) Six-week-old C57/BL6 mice were infected with the same doses of PR8-M1 and PR8-M1-K102R viruses. Body weight (C) and survival (D) were monitored daily for 2 weeks (*n* = 10).

Next, we inoculated mice with WT and M1-K102R mutant H5N6 or PR8 viruses at a dose of 2× MLD50. The results demonstrated that H5N6-M1-K102R and PR8-M1-K102R viruses were remarkably less pathogenic than their respective WT viruses. Mice infected with a H5N6-M1 virus displayed severe weight loss, disease, and 60% mortality, while those infected with the same dose of H5N6-M1-K102R virus exhibited 30% mortality (Fig. 8A and B). Similarly, mice infected with PR8-M1-K102R virus exhibited slight weight loss and 40% mortality, while those infected with PR8-M1 virus displayed severe disease and 80% mortality (Fig. 8C and D). Taken together, these data suggested that a mutation at position K102 of M1 impaired the pathogenicity and lethality of PR8 (H1N1) and JX (H5N6) influenza viruses in mice.

### Gross pathology, histopathology, and viral loads in trachea and lungs.

**FIG 9:**
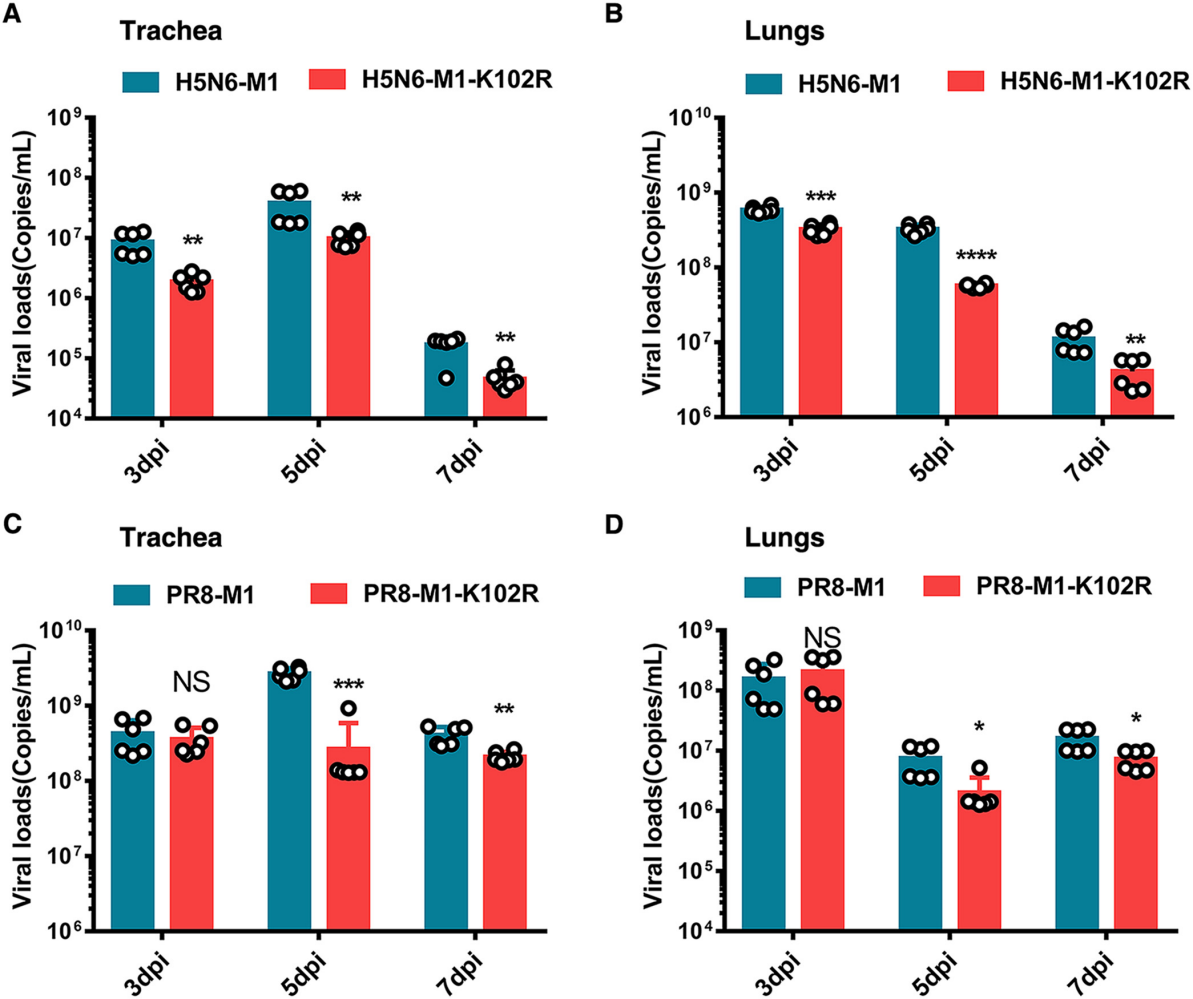
Viral loads of C57/BL6 mice after influenza virus infection. Six-week-old C57/BL6 mice were infected intranasally with WT and M1-K102R mutant H5N6 or PR8 viruses at a dose of 2× MLD50. (A, B) Viral RNA copies were determined by qRT-PCR at 3, 5, and 7 dpi in the trachea (A) and lung (B) of mice infected with H5N6-M1 or H5N6-M1-K102R virus. (C, D) Viral RNA copies were determined by qRT-PCR at 3, 5, and 7 dpi in the trachea (C) and lung (D) of mice infected with PR8-M1 or PR8-M1-K102R virus. Error bars, mean ± SD of three experiments. All comparisons were done by two-tailed Student’s *t* test; *, *P* < 0.05; **, *P* < 0.01; ***, *P* < 0.001.

The pulmonary gross pathology was consistent with IAV respiratory disease at 3, 5, and 7 days postinfection (dpi) in lung samples from mice infected with each virus (see Fig. S9A and B in the supplemental material). In particular, mice infected with H5N6 virus exhibited obvious lung lesions at 3 dpi. Generally, the pulmonary gross pathology caused by M1-K102R mutant virus infection was reduced compared with that caused by WT virus. At 5 dpi, hematoxylin and eosin (H&E) staining revealed moderate to severe bronchiolar necrosis, pulmonary edema, and inflammatory cell infiltrates in lung tissues from mice infected with H5N6-M1 or PR8-M1 virus, while lung lymphoid tissue infiltration was restricted in mice infected with H5N6-M1-K102R or PR8-M1-K102R virus (Fig. S9C and D). Furthermore, mice infected with M1-K102R mutant viruses exhibited lower viral loads in the trachea and lungs than those infected with WT viruses (Fig. 9A to D). In addition, we sequenced virus from mouse lungs at 7 dpi to test whether the M1 K102R mutation would revert after replication *in vivo*. Results showed that the K102 site of M1 did not undergo reverse mutation (data not shown). Overall, these results further supported that the M1-K102 site mutation impaired the virulence of the influenza virus in mice.

## DISCUSSION

In this study, we identified PSMD12 as a novel interacting partner of M1 protein of the influenza virus in avian DF1 cells through IP/MS. Further study demonstrated that PSMD12 positively regulated the replication of IAV in avian DF1 and human A549 cells. Interestingly, our data showed that PSMD12 promoted M1-M2 VLP egress. Meanwhile, PSMD12 promoted K63-linked ubiquitination of M1 at the K102 site and M1-K102 mutation affected the formation of supernatant M1-M2 VLPs. To further clarify the role of the mutation of M1 at K102, we constructed M1-K102R mutant strains of H5N6 and PR8 viruses. The M1-K102 site mutation interfered with the budding of the mutant viruses and significantly affected the virulence of H5N6 and PR8 viruses in mice. There is no doubt that M1 K102 is an important site for virus replication. On the host side, we demonstrated that PSMD12 regulated virus budding and morphology of H5N6 virus. In summary, our results demonstrate that PSMD12 and PSMD12-mediated ubiquitination of M1 at K102 contribute to influenza virus budding and release, which is a novel mechanism by which the influenza virus hijacks a host factor to benefit its replication.

Ubiquitination is a posttranslational modification that creates versatility in intracellular signaling. Ubiquitin has seven lysine residues, namely, K6, K11, K27, K29, K33, K48, and K63, which result in the formation of polyubiquitin chains, although linkages generally occur via K48 or K63 (20, 21). K48-mediated polyubiquitination promotes the degradation of ubiquitinated proteins of the 26S proteasome (22). whereas K63-linked polyubiquitination results in nonproteasomal modifications, such as subcellular localization or protein-protein interactions (23). PSMD12 is a 26S proteasome regulatory subunit (aka RPN5). Unexpectedly, our results indicated that PSMD12 triggers K63-linked ubiquitination of M1 without affecting K48-linked ubiquitination of M1. The ubiquitination of proteins is carried out by the successive action of ubiquitin-activating (E1), ubiquitin-conjugating (E2), and ubiquitin-protein ligase (E3) enzymes (24). The final step of the ubiquitination process requires a substrate-specific interaction partner, an E3 ubiquitin ligase. As reported previously, the E3 ubiquitin-protein ligase MARCH8 reduces influenza A virus release by promoting M2 ubiquitination at the K78 site of the influenza virus (24). However, the current results do not confirm PSMD12 as an E3 ubiquitin ligase nor as an adaptor to recruit an E3 ubiquitin ligase to M1 for ubiquitination. Mahesutihan et al. provide evidence that E3 ubiquitin ligase AIP4 promoted K48-linked ubiquitination of M1 at the K102 and K104 site (25). However, it does not appear to synergize with PSMD12 to promote K63-linked ubiquitination of M1 (Fig. S10). K48-linked and K63-linked ubiquitination at the M1 K102 site may be mediated by two different E3 ubiquitin ligases. Which E3 ubiquitin ligase modifies M1 mediated by PSMD12 deserves further investigation.

For enveloped viruses, host factors are required in the multistep process of virion assembly and release and are commonly hijacked by the virus to execute membrane remodeling and budding (26). The L domain has been reported to exist in the matrix proteins of many enveloped viruses, and it is critical for the budding of virions from the plasma membrane. In influenza virus, the helix six (H6) domain encompassing the nuclear localization signal (NLS) motif in the matrix protein M1 has been shown to act as the L domain (27), which includes the motif 101-RKLKR-105, and has been demonstrated as a nuclear localization signal. Moreover, this domain is also associated with functions such as binding to the ribonucleoprotein genome segments (RNPs), membrane association, interaction with the viral nuclear export protein, and virus assembly. The L domain has been reported to work through recruiting a number of host proteins at the budding site, which are required to initiate the budding process (28) and release the virion particles. As indicated earlier, viral L domains bind to components of the endosomal sorting complex required for transport (ESCRT) machinery, which is typically involved in the formation of vesicles into multivesicular bodies, such as Tsg101, Nedd4, ubiquitin ligases, and others (29–32). In the present study, we provide evidence that PSMD12 promotes the egress of M1-M2 VLPs and demonstrate that the K102 site of M1 is crucial to the formation of M1-M2 VLPs. Furthermore, knockdown of PSMD12 or mutation of the M1 K102 site results in the defective release of the virus. Notably, the M1 K102 site is contained in the L domain as described above. Therefore, the associations between PSMD12 and ESCRT machinery and the E3 ubiquitin ligases associated with L domain are worthy of attention in future studies.

Influenza A virus is found in a morphologically heterogeneous filamentous form that predominates in human isolates, with virions having a uniform diameter of ~80 to 100 nm but lengths ranging from ~100 nm to several microns (33). After many passages of chick embryos or cells, almost all retained virions are spherical, and this morphologic change was associated with an increase in viral titer (34, 35). Several studies have shown that M1 is an important determinant of influenza virus morphology. Elleman et al. demonstrated that amino acids at positions 41, 95, and 218 of M1 had a great influence on the filamentous phenotype (36). Wu et al. reported that wild-type influenza (WSN) virus-infected Huh7 cells released many more spherical viral particles than the M1-K242 mutant virus-infected cells (37). In this study, mutation of the PSMD12-mediated M1 ubiquitination site (i.e., K102 site) caused the H5N6 virus to change from spherical to filamentous. Importantly, PSMD12 regulates H5N6 virus proliferation that is dependent on the M1 K102 site. In turn, knockdown of PSMD12 changed the shape of the virus from spherical to bacilliform or even filamentous. This evidence suggests that ubiquitination at K102 of M1 plays an important role in the regulation of virus morphology. The Udorn (a filamentous influenza virus) M1 protein likely confers the filamentous phenotype by stabilizing specific helical contacts in the matrix layer (38). Mutation at the M1 K102 site affects virus budding and morphology. As explained by Burleigh et al., length variation may reflect a competition between the extension of the M1 helix and the completion of budding and release (38). We speculate that the change of virus morphology may be related to the restriction of assembly and budding. The explanation for these results needs to be further explored in the future.

Efficient multicycle replication, including virus release and infection of new cells, is obligatory for virus survival and pathogenesis. In fact, mutation of M1 at K102 caused a decrease in the pathogenicity of both H5N6 and PR8 viruses in mice. Severe lung injury and death during IAV infection result from an exuberant host inflammatory response (39). There is a modest reduction in pulmonary virus titers of the mutant virus but a notable reduction in inflammatory responses. This finding may be due to the reduced viral titer of the mutant virus attenuating the overactivation of the host immune system, resulting in a greatly weakened inflammatory cascade. Recent studies highlighted that M1 is one of the slowest-evolving proteins encoded by the influenza virus genome (40, 41). While there are differences in M1 gene evolutionary rates between viruses infecting different host species, IAV strains sampled globally in humans and across a range of other host species exhibit over 95% amino acid sequence identity for the M1 protein (40–43). Therefore, targeting M1 might provide an important future research direction for anti-influenza virus drugs. Notably, K102 is a highly conserved amino acid site in the M1 sequence of the influenza virus. On the host side, this study provides evidence that PSMD12 positively regulates the proliferation of influenza virus *in vitro*. Unfortunately, the regulatory effect of PSMD12 on influenza virus was not verified *in vivo* due to the maldevelopment caused by the PSMD12 deletion (44).

In summary, we identified PSMD12 as a novel host factor involved in IAV replication by mediating K63-linked ubiquitinated M1 at the K102 site to affect viral release. This study not only advances the mechanistic understanding of influenza viral protein regulation by host proteins but also paves the way for a more sophisticated therapeutic approach to combat influenza infection.

## MATERIALS AND METHODS

### Ethics statement.

This study was conducted in strict accordance with the recommendations provided in the Guide for the Care and Use of Laboratory Animals of the Ministry of Science and Technology of the People’s Republic of China. All animal experiments were approved by the Research Ethics Committee, Huazhong Agricultural University, Hubei, China (approval no. HZAUMO-2019-080).

### Cells and viruses.

The chicken embryo fibroblast DF1 cell line, human embryonic kidney 293T cells (HEK293T), human cervical cancer cell line (HeLa), and Madin-Darby canine kidney (MDCK) cells were maintained in Dulbecco’s modified Eagle’s medium (DMEM) (Gibco, Grand Island, NY) supplemented with 10% fetal bovine serum (FBS) (PAN-Biotech, Aidenbach, Germany). Adenocarcinomic human alveolar basal epithelial cells (A549) were maintained in F12 media supplemented with 10% FBS. Primary chicken embryo fibroblast (CEF) cells were isolated from 9-day-old embryonated eggs. Cells were cultured at 37°C in a humidified atmosphere containing 5% CO_2_. The IAV strain used in this study was the A/duck/Hubei/WH18/2015(H5N6) (JX) (GenBank accession no. KX652135) strain isolated in our laboratory from an infected duck lung. Recombinant viruses were generated on genetic backgrounds of either wild-type (WT) A/Puerto Rico/8/34 (H1N1) (PR8) or H5N6 (JX) virus using an eight plasmid-based reverse genetics system, as described previously (45).

### Transfections.

Transfections were performed using Lipofectamine 2000 (Invitrogen, Carlsbad, CA) at a 2:1 Lipofectamine/DNA or RNA ratio in Opti-MEM serum-free medium (Invitrogen) according to the manufacturer’s protocol.

### Coimmunoprecipitation (co-IP) assay.

Cells were transfected with the indicated plasmids. Whole-cell lysates from cells were prepared as reported previously (46). Coimmunoprecipitation (co-IP) was performed using anti-Flag or anti-HA antibody-conjugated magnetic beads, and the immune complexes were captured on magnetic beads (MedChemExpress, Monmouth Junction, NJ). Subsequently, the beads were washed thrice with lysis buffer and eluted with 1× sodium dodecyl sulfate (SDS) loading buffer. Samples were evaluated using Western blotting.

### Mass spectrometry analysis.

To obtain M1-associated host factors, we performed immunoprecipitation-mass spectrometry (IP/MS) experiments. DF-1 cells were seeded into six-well plates and transfected with pCAGGS-HA-M1 or pCAGGS-HA empty vectors for 24 h. DF-1 cells were subsequently subjected to phosphate-buffered saline (PBS) wash and radioimmunoprecipitation assay (RIPA) lysis buffer (Beyotime, China). They were then incubated with HA-labeled agarose beads (MedChemExpress, Monmouth Junction, NJ) for 8 h and rotated at 4°C. Bead-bound immune complexes were washed 5 times and collected for subsequent western imprinting and silver staining assay. Protein bands were excised and subjected to tryptic digestion. After reduction and alkylation, trypsin (mass ratio, 1:50) was added and hydrolyzed at 37°C for 20 h. After desalination, the enzymatic hydrolysis product was lyophilized and redissolved in 0.1% formic acid solution. The tandem mass spectrometry (MS/MS) signals were processed against the Uniprot Gallus protein database (36,624 sequences) using the Mascot 2.2 algorithm with the following parameters: variable modifications, oxidation (Met), N-acetylation, pyroglutamination (Gln); maximum missed cleavages, 2; peptide mass tolerance, 100 ppm; and MS/MS tolerance, 0.5 Da. The criterion used for protein identification was based on having at least one MS/MS data signal with Mascot scores that exceeded the thresholds (*P* < 0.05).

### Generation of PSMD12-knockdown A549 cells.

A549 cells stably expressing PSMD12 shRNA or control nontarget shRNA were established using a vector-based shRNA technique, as described previously (37). The PLKO.1 vector psPAX2 and pMD2.G were cotransfected into HEK293T cells. The cell supernatant was harvested 48 h posttransfection. Subsequently, A549 cells were incubated with the viral supernatant. Finally, monoclonal cells were sorted using flow cytometry and seeded into a 96-well plate to generate clonal cell lines. PSMD12 knockdown in A549 cells was confirmed via Western blotting.

### Cytotoxicity assay.

The viability of DF1 cells after transfection with siRNA was measured using a CCK-8 assay according to the manufacturer’s instructions (Bimake, Shanghai, China) (47). Briefly, cells were seeded into the 96-well plate and transfected with siRNA when the cell density reached approximately 80%; the CCK-8 reagent was added 24 h after transfection. After 2 h of incubation at 37°C, the absorbance of samples was measured at 450 nm. The viability of A549 cells after sh-PSMD12 transfection was measured using the same method.

### Viral plaque and TCID_50_ assays.

The viral plaque and 50% tissue culture infectious dose (TCID_50_) assays were performed as described previously (48). Briefly, confluent MDCK cells in 12-well plates were inoculated with the appropriately diluted virus in DMEM and incubated at 37°C for 1 h. The cells were washed twice with phosphate-buffered saline (PBS) (HyClone) and then were overlaid with a mixture of 2.0% agarose and double-strength DMEM (Gibco). After incubation at 37°C for 72 h, cells were fixed and stained with 0.1% crystal violet, and the plaques were counted. The TCID_50_ assay was carried out in 96-well cell plate.

### Immunofluorescence staining and microscopy.

Immunofluorescence staining and microscopy were performed as described previously (49). Briefly, cells were fixed with 4% paraformaldehyde at room temperature for 10 min followed by treatment with 0.1% (vol/vol) Triton X-100 for 10 min. The cells were incubated in 2% (vol/vol) bovine serum albumin (BSA) for 1 h at room temperature. Subsequently, the cells were incubated with the designated primary antibody for 2 h and then with the appropriate Alexa Fluor-conjugated secondary antibody for 1 h. Finally, the cells were stained with 4′,6-diamidino-2-phenylindole (DAPI) to visualize DNA. The cells were observed, and images were obtained using an LSM880 confocal microscope (Carl Zeiss, Wetzlar, Germany).

### RNA isolation and qRT-PCR.

For reverse transcription-quantitative PCR (qRT-PCR), RNA was extracted using TRIzol (Thermo Fisher Scientific, Waltham, MA) as described previously. Immediately thereafter, RNA was transcribed using reverse transcriptase (AMV XL; TaKaRa Bio, Tokyo, Japan). Real-time PCR was performed using FastStart Universal SYBR green master mix (Roche, Basel, Switzerland) on a Vii7A real-time PCR system (ABI, Foster City, CA). The sequences of the primers used for qRT-PCR are listed in Table S3 in the supplemental material.

### Kinetics of virus growth.

MDCK/DF1/CEF/A549 cells were washed with DMEM (or Ham’s/F-12) and infected with the indicated virus at an MOI of 0.05. The inoculum was removed after 1 h of virus adsorption. Cells were then washed with DMEM (or Ham’s/F-12) and cultured in minimal essential medium (PR8 virus-infected MDCK cells were grown with minimal essential medium containing 4 μg/mL *N*-tosyl-l-phenylalanine chloromethyl ketone [TPCK]-trypsin, and PR8 virus-infected A549 cells were grown with minimal essential medium containing 2 μg/mL TPCK-trypsin). The supernatants of infected cells were collected at different time points after infection and added to MDCK cells. Viral titers were determined by calculating log_10_TCID_50_/mL using the Karber method (50).

### NA activity assay.

NA enzymatic activity assay was performed as described previously (51), and samples were analyzed using a neuraminidase assay kit (Beyotime, China). Briefly, wild-type and mutated influenza viruses were calibrated with same TCID_50_ titer by diluting the virus samples and transferred into 1.5‐mL Eppendorf tubes. Then, virus (10 μL) was mixed with 70 μL detection buffer, 10 μL NA fluorogenic substrate, and 10 μL double-distilled water. Cleavage of the substrate by NA activity produced a fluorescence that emitted an emission wavelength of 450 nm with an excitation wavelength of 320 nm. Fluorescence was monitored using a multifunctional microplate reader (Tecan).

### Transmission electron microscopy.

CEF cells were infected with H5N6-M1 (H5N6-WT) or H5N6-M1-K102R (mutant H5N6) virus for 10 h at an MOI of 0.1. Subsequently, the cells were fixed with 2.5% glutaraldehyde for 2 h at room temperature, harvested and fixed with 2.5% glutaraldehyde on ice for 2 h, followed by fixation in 2% osmium tetroxide. Finally, the cells were dehydrated with sequential washes in 50%, 70%, 90%, 95%, and 100% ethanol. Areas containing cells were block mounted and sliced thinly. TEM images of the products were performed by using transmission electron microscopy (Japan, NTC, JSM-6390LV).

### Virus-like particle (VLP) preparation and analysis.

HEK293T cells (1 × 10^6^ cells) were seeded in 6-cm-diameter dishes and grown in DMEM-10% FBS for 24 h. To generate influenza virus-like particles (VLPs), the cells were transfected with the appropriate plasmid DNA. At 6 h posttransfection, the transfection medium was replaced with DMEM-10% FBS. At 24 h posttransfection, the culture medium was harvested, and cellular debris was pelleted by centrifugation at 2,000 × *g* for 10 min. The culture medium was then centrifuged at 100,000 × *g* for 3 h at 4°C. The pellet was resuspended in sodium dodecyl sulfate (SDS) loading buffer (50 mM Tris [pH 6.8], 100 mM dithiothreitol, 2% SDS, 0.1% bromophenol blue, and 10% glycerol), boiled for 10 min, and analyzed by SDS-PAGE on a 12% polyacrylamide gel.

### Pathogenicity study in mice.

To determine the 50% median lethal dose (MLD50), 6-week-old female C57/BL6 mice were anaesthetized lightly with CO_2_ and inoculated intranasally with 50 μL of 10-fold serial dilutions of PR8-M1 (PR8-WT) or H5N6-M1 (H5N6-WT) virus (*n* = 8). Mice were monitored for weight loss and mortality daily for 14 days. Mice that lost ≥30% of their initial weight were euthanized humanely. MLD50 values were calculated using the Karber method. To compare the virulence of wild and mutated viruses *in vivo*, C57/BL6 mice were inoculated intranasally with the indicated doses of PR8-M1, PR8-M1-K102R, H5N6-M1, or H5N6-M1-K102R virus diluted in PBS (*n* = 10) and euthanized 3, 5, and 7 days postinfection (dpi). Viral RNA copies in the lung and trachea were determined through qRT-PCR.

### Histopathology and immunohistochemistry.

The trachea and lungs obtained from individual mice euthanized at various time points were fixed in 10% neutral buffered formalin. Sections were stained with hematoxylin and eosin (H&E) and examined using light microscopy.

### Statistical analysis.

Statistical analyses were performed using Premier 6.0 software (GraphPad Prism, San Diego, CA) by applying unpaired Student’s *t* test or one-way analysis of variance (ANOVA). Two-way ANOVA was used when appropriate. *P* values of <0.05 were considered statistically significant, with the following symbols: *, *P* < 0.05; **, *P* < 0.01; ***, *P* < 0.001; and ****, *P* < 0.0001. The number of mice in each group and specific details on statistical tests are described in the figure legends. Venn diagrams were generated using the Venn Diagram Plotter (http://omics.pnl.gov/software/venn-diagram-plotter). Pearson correlation coefficients were determined for molecular colocalization in confocal microscopy images using Coloc 2 of Fiji.

### Laboratory facility.

All experiments involving live viruses were performed in a biosafety level 3 (BSL3) facility at Huazhong Agricultural University in accordance with the institutional biosafety manual. The animals were housed in negative-pressure isolators with high-efficiency particulate air filters in the BSL3 facility.
