# Supplementary material for: PSMD12-Mediated M1 Ubiquitination of Influenza A Virus at K102 Regulates Viral Replication
Source: J Virol. 2022 Jul 21;96(15):e00786-22. doi: 10.1128/jvi.00786-22 (PMC9364790; doi:10.1128/jvi.00786-22)
Supplement: Supplemental file 1 — Supplemental material. Download jvi.00786-22-s0001.pdf, PDF file, 1.1 MB [file jvi.00786-22-s0001.pdf]

Supplementary Materials for

PSMD12-mediated M1 ubiquitination of influenza A virus at K102 regulates viral replication

Xianfeng Hui[ab], Lei Cao[ab], Ting Xu[ab], Lianzhong Zhao[ab], Kun Huang[ab], Zhong Zou[abc], Peilei Ren[ab], Haiying Mao[ab], Ying Yang[abc],Shuo Gao[ab], Xiaomei Sun[ab], Xian Lin[ab*] and Meilin Jin[abcd*]

[a]*State Key Laboratory of Agricultural Microbiology, Huazhong Agricultural University, Wuhan, China*

[b]*College of Animal Medicine, Huazhong Agricultural University, Wuhan, China*

[c]*Key Laboratory of development of veterinary diagnostic products, Ministry of Agriculture, Wuhan, Hubei, People's Republic of China*

[d]*The Cooperative Innovation Center for Sustainable Pig Production, Wuhan, China*

*** Correspondence:** jinmeilin@mail.hzau.edu.cn (MJ); yoya12@163.com (XL)

This PDF file includes:

Supplementary Table, Figures and Figure legends

Supplementary Table

Table S1. Candidate Host proteins for Further Analysis

Gene ID	Gene abbreviation	protein name	NCBI accession number
429558	HIST1H2B5L	histone cluster 1, H2B-V-like (similar to human histone cluster 1, class H2B genes)	XM_004934773
395853	HSPA8	heat shock 70kDa protein 8	NM_205003
420039	KRTC42L	keratin, type I cytoskeletal 42-like	-
427882	HISTH1	histone H1	NM_001396439
395855	CALM2	calmodulin 2	NM_205005
423504	HSP70	heat shock 70kDa protein 2	NM_001006685
374193	GAPDH	glyceraldehyde-3-phosphate dehydrogenase	NM_204305
424115	DNAJC10	DnaJ heat shock protein family (Hsp40) member C10	XM_046943855
419963	MYO1D	myosin ID	XM_040653400
**417425**	**PSMD12**	**proteasome 26S subunit, non-ATPase 12**	**NM_001316341**
418016	RPL3	ribosomal protein L3	NM_001006241
420001	RPL23	ribosomal protein L23	NM_001321599
418710	RPL31	ribosomal protein L31	NM_001277755
416275	RPS14	ribosomal protein S14	XM_015293785
770103	TPM3	tropomyosin 3	NM_001245927
100859627	HNRNPA3	heterogeneous nuclear ribonucleoprotein A3	XM_025152378
-	A0A1D5PQ92	Uncharacterized protein	-
-	A0A1D5PK18	Uncharacterized protein	-
-	TBA1	Tubulin alpha-1 chain	-

Table S2. siRNA target PSMD12 in this study.

Name	Sense sense（5'-3'）	antisense（5'-3'）
siPSMD12-1	GCGUAUCUUAGUGGCU AUATT	UAUAGCCACUAAGAUACG CTT
siPSMD12-2	**GCAGAUGAGGCUCUGU CUATT**	**UAGACAGAGCCUCAUCU GCTT**
siPSMD12-3	CCACACAUCUCAUUGCC AATT	UUGGCAAUGAGAUGUGU GGTT
shRNA-1	Target sequence (DNA): GGTTACCGAAGGCAAGATTTA	
shRNA-2	Target sequence (DNA): GCACAGCTTCTGGATCTATCT	
shRNA-3	Target sequence (DNA): **GCACTACAGAGCAATATATGA**	

Table S3. The main primers used in this study.

Name	Sequence（5'-3'）
K21R-F	CATCCCATCAGGCCCCCTCAGAGCCGAG
K21R-R	GTTTCTGCGCGATCTCGGCTCTGAGGGG
K35R-F	GATGTGTTTGCAGGAAGAAACGCTG
K35R-R	CCTCGAGATCAGCGTTTCTTCCTG
K98R-F	GGGCAGTTAGGCTATATAAGAAGCTG
K98R-R	CTTATATAGCCTAACTGCCCTATCC
K101R-F	GTTAAGCTATATAGGAAGCTGAAAAG
K101R-R	CTCTTTTCAGCTTCCTATATAGC
K102R-F	GCTATATAAGAGGCTGAAAAGAG
K102R-R	CTCTTTTCAGCCTCTTATATAGC
K113R-F	GGAGCTAGGGAGGTCGCACTCAGTTAC
K113R-R	GTGCGACCTCCCTAGCTCCATGG
K187R-F	CTACAGCTAGGGCTATGGAGCAGATGG
K187R-R	CCATAGCCCTAGCTGTAGTGCTGGC
K242R-F	CCTACCAGAGACGAATGGGAGTGCAG
K242R-R	CCCATTCGTCTCTGGTAGGCCTGC
K252R-F	GATGCAGCGATTCAGGTGAGGATC
K252R-R	GCCCGGGATCCTCACCTGAATCGC
qRT-PCR-IAV-M1-F	CTTCTAACCGAGGTCGAAACG
qRT-PCR-IAV-M1-R	GGCATTTTGGACAAAKCGTCTA
Uni12 primer	AGCAAAAGCAGG

Supplementary Figures and Figure legends

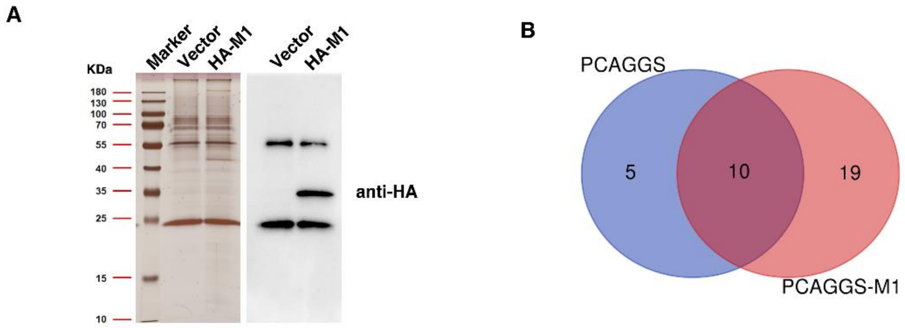

Supplementary Figure 1. Identification of influenza virus M1-associated factors by immunoprecipitation-mass spectrometry (IP/MS). (A) The coimmunoprecipitated proteins were separated and visualised by silver staining (left) and western blotting (right). (B) Venn diagram.

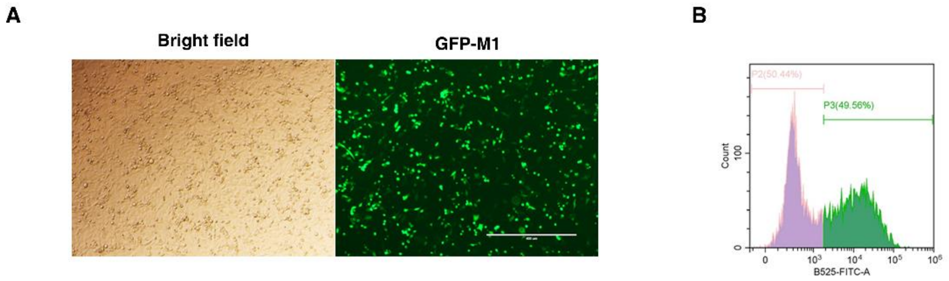

Supplementary Figure 2. Transfection efficiency of Lip2000 reagent on DF1 cells. (A) Fluorescence observation 24 hours after transfection of pEGFP-M1 plasmid into DF1 cells using Lip2000 reagent. (B) The transfection efficiency was determined by flow cytometry analysis.

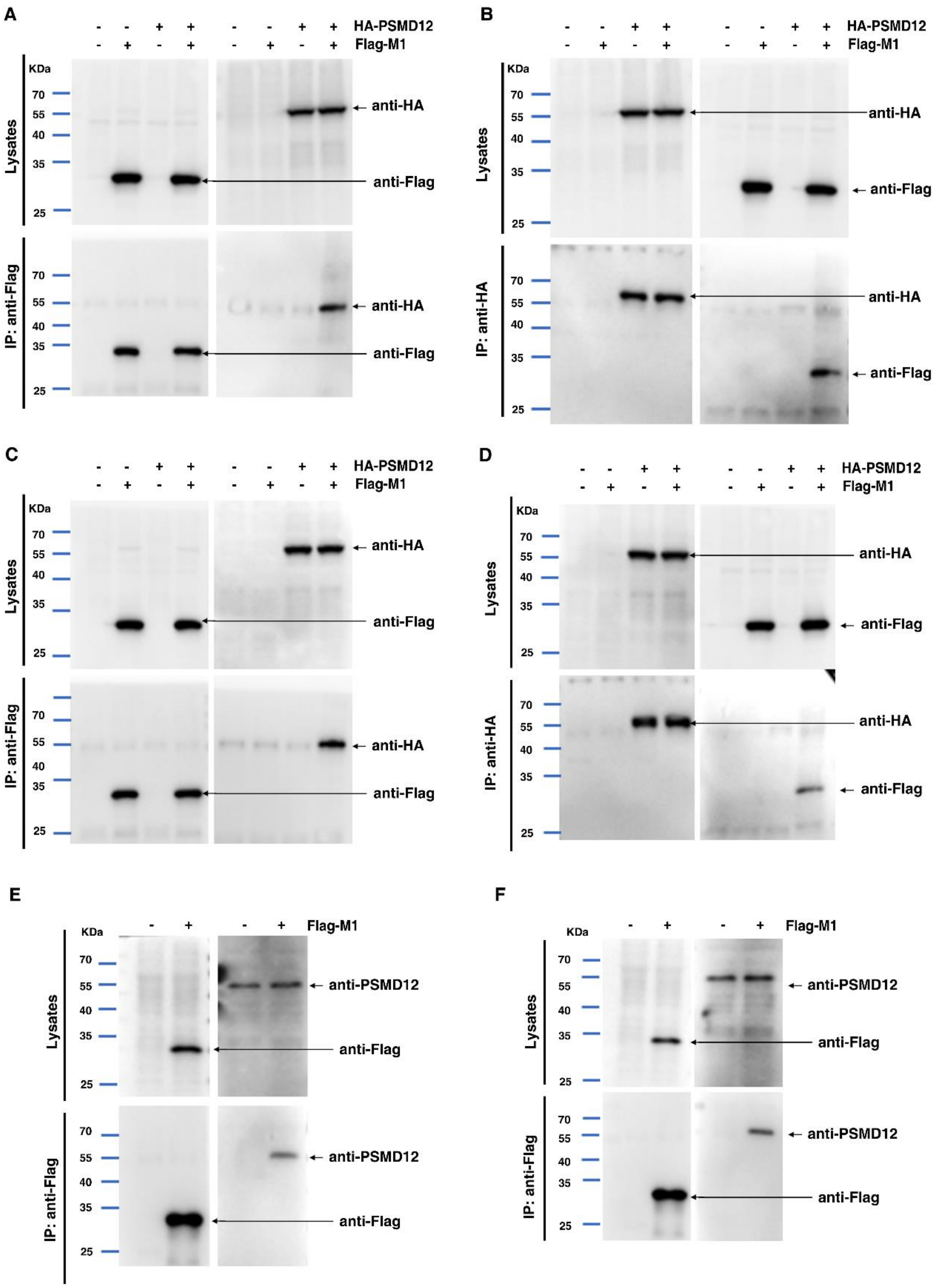

Supplementary Figure 3. Full western blot figure reference to Figure 3. (**A**, reference to Figure 3A) DF1 cells were transfected with plasmids encoding HA-PSMD12 and Flag-M1. The lysates were subjected to anti-Flag IP and analysed via western blotting (WB). (**B**, reference to Figure 3B) DF1 cells were transfected with plasmids encoding HA-PSMD12 and Flag-M1. The lysates were subjected to anti-HA IP and analysed via WB. (**C**, reference to Figure 3F) HEK293T cells were transfected with plasmids encoding HA-PSMD12 (human source) and Flag-M1. The lysates were

subjected to anti-Flag IP and analysed via WB. (**D**, reference to Figure 3G) HEK293T cells were transfected with plasmids encoding HA-PSMD12 and Flag-M1. The lysates were subjected to anti-HA IP and analysed via WB. (**E**, reference to Figure 3C) DF1 cells were transfected with a plasmid encoding Flag-M1, and the lysate was subjected to IP and analysed for endogenous PSMD12 via WB. (**F**, reference to Figure 3H) HEK293T cells were transfected with a plasmid encoding Flag-M1, and the lysate was subjected to IP and analysed for endogenous PSMD12 via WB.

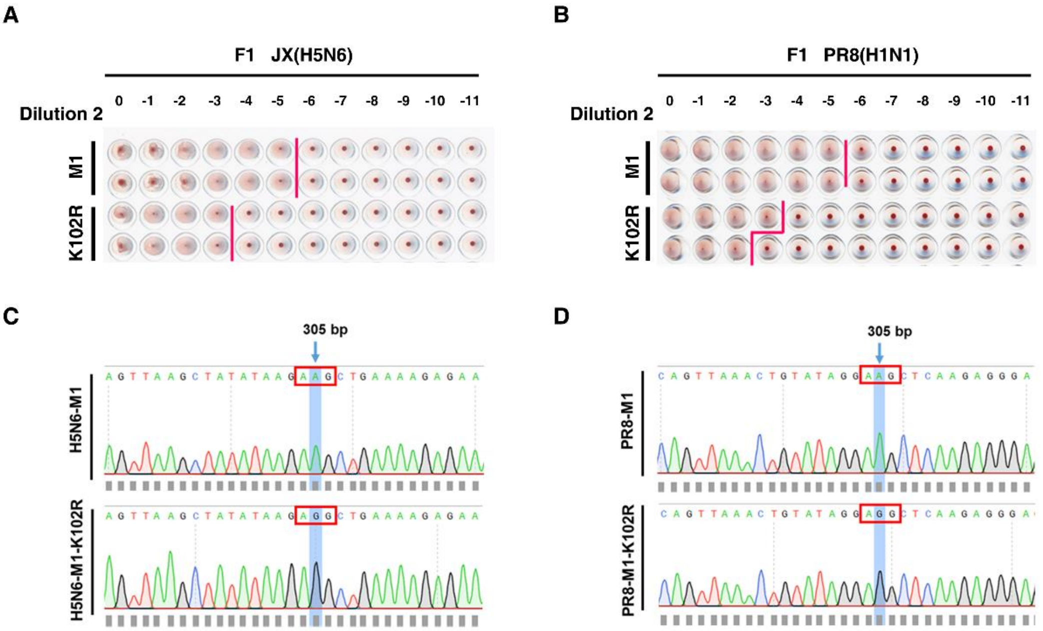

Supplementary Figure 4. H5N6 and PR8 M1-K102R mutant viruses obtained by infectious cloning. (A) Hemagglutination test was performed on the allantoic fluid of chicken embryos infected with the first-generation H5N6 virus plasmid recombinant virus. (B) Hemagglutination test was performed on the allantoic fluid of chicken embryos infected with the first-generation PR8 virus plasmid recombinant virus. (C) H5N6 and it mutants viral RNA sequence was sequenced by Sanger method. (D) PR8 and it mutants viral RNA sequence was sequenced by Sanger method.

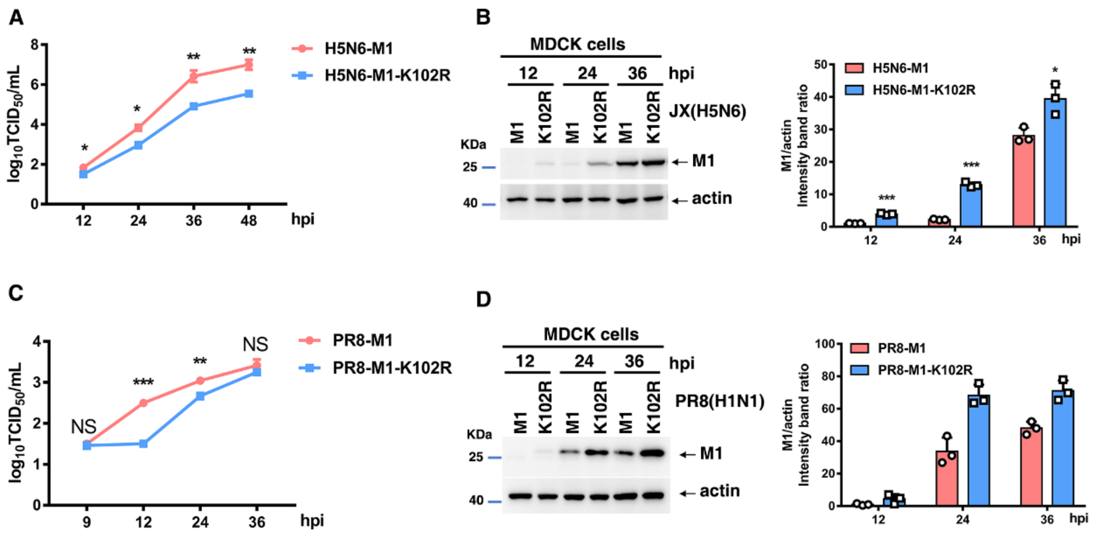

Supplementary Figure 5. Mutation at the M1 K102 site alters viral replication in cells. (A, B) MDCK cells were infected with H5N6-M1 or H5N6-M1-K102R virus, the supernatant was collected at different time points for $TCID_{50}$ measurement, and the cell lysates were analysed by western blotting (WB). (C, D) MDCK cells were infected with PR8-M1 or PR8-M1-K102R virus, the supernatant was collected at different time points for TCID50 measurement, and the cell lysates were analysed by WB. Error bars, mean ± SD of three experiments. All by two-tailed Student's t test; *p < 0.05; **p < 0.01; ***p < 0.001.

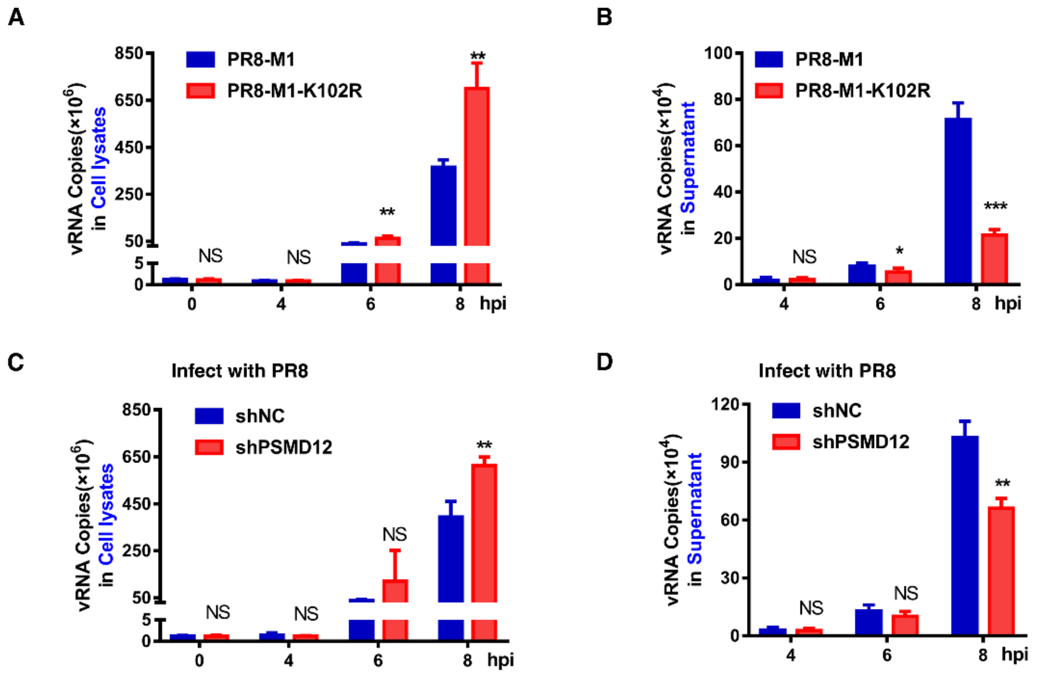

Supplementary Figure 6. M1 K102 mutation or knockdown PSMD12 restricts virus release. (A, B) A549 cells were infected with PR8-M1 or PR8-M1-K102R virus (MOI of 5), the cell lysates (A) and supernatant (B) were collected at different time

points and analysed vRNA of M1 by qRT-PCR. (C, D) A549 cells were infected with PR8-M1 virus at MOI=5. The cell lysates (C) and supernatant (D) were collected at different time points and analysed vRNA of M1 by qRT-PCR. All by two-tailed Student's t test; *p < 0.05; **p < 0.01; ***p < 0.001.

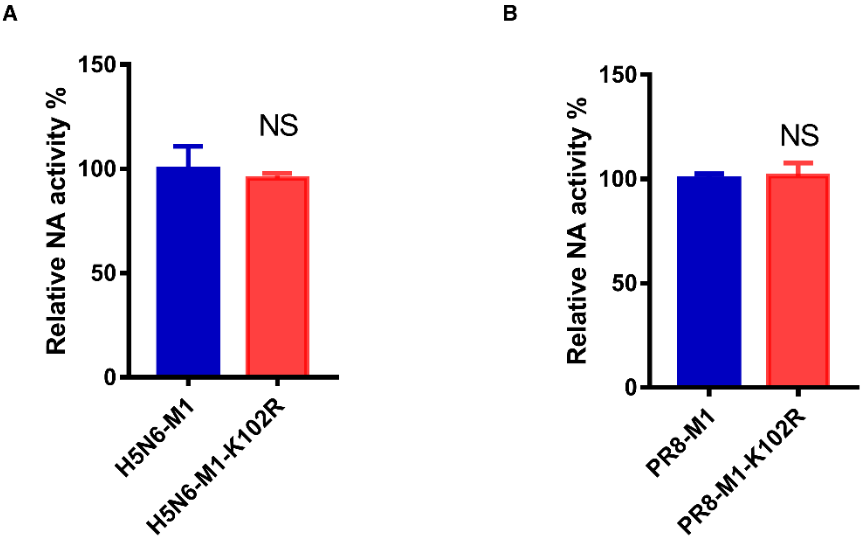

Supplementary Figure 7. Detection of NA activity of influenza virus. (A, B) The NA activity of H5N6 (A) and PR8 (B) viruses was determined as described in materials and methods (NA activity assay). The results are displayed as mean ± SD of three independent experiments. The comparisons were performed with t tests.

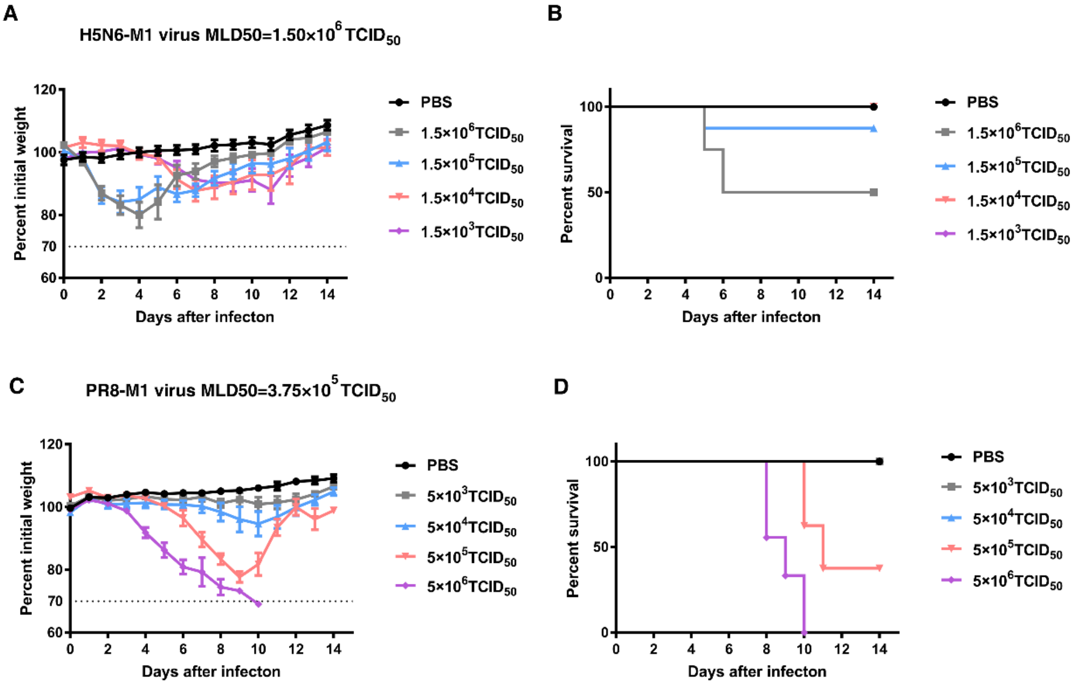

Supplementary Figure 8. MLD50 dose of PR8 and H5N6 influenza viruses. (A, B)

Six-week-old C57/BL6 mice were intranasally infected with 10-fold serially diluted -H5N6-M1 virus. Body weight (A) and survival (B) were monitored daily for two weeks (n=8). (C, D) Six-week-old C57/BL6 mice were intranasally infected with 10-fold serially diluted PR8-M1 virus. Body weight (C) and survival (D) were monitored daily for two weeks (n=8).

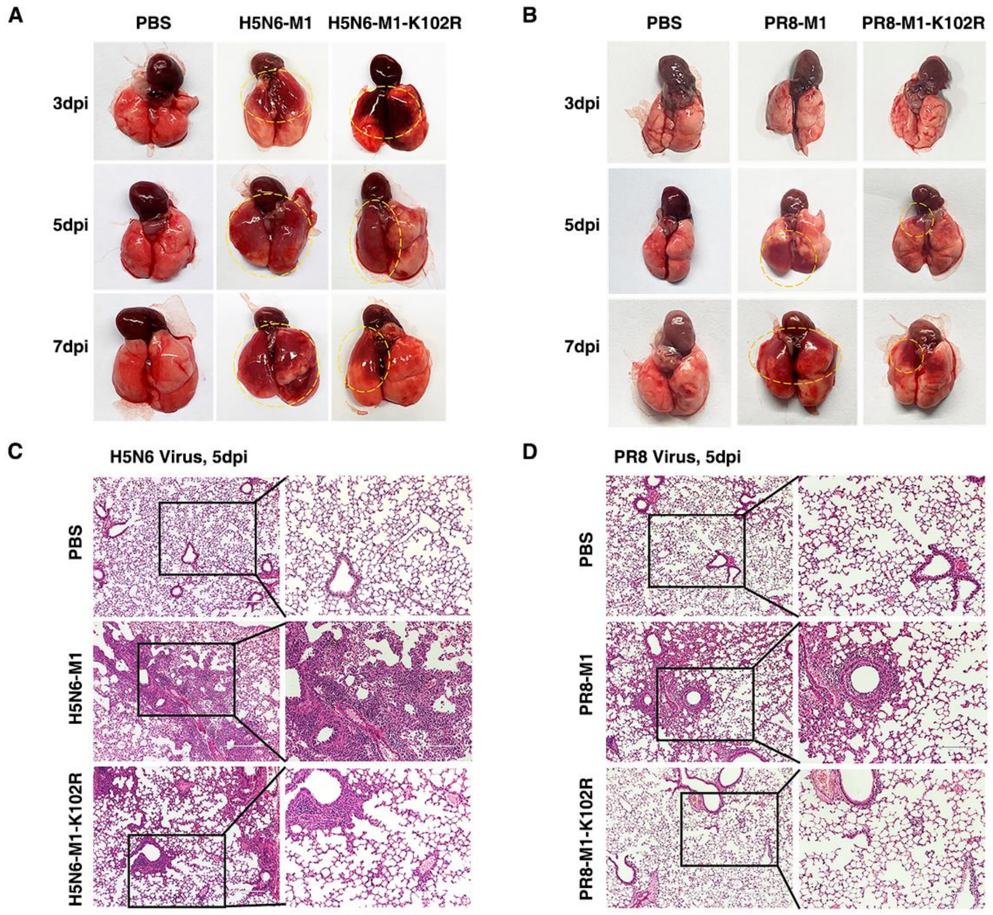

Supplementary Figure 9. Gross pathology and histopathology. Six-week-old C57/BL6 mice were intranasally infected with WT and M1-K102R mutant H5N6 or PR8 viruses at a dose of 2×MLD50. (A, B) Macroscopic appearance of lung tissue of C57/BL6 mice at 3, 5, and 7 dpi with H5N6 (A) or PR8 (B) virus. Post-mortem findings revealed focal dark red lesions (circled). (C, D) Lung tissues of C57/BL6 mice challenged with H5N6-M1 or H5N6-M1-K102R virus (C) and PR8-M1 or PR8-M1-K102R virus (D) at 5 dpi. H&E staining shows inflammatory cell infiltration and moderate interstitial pneumonia with thickened alveolar septa.

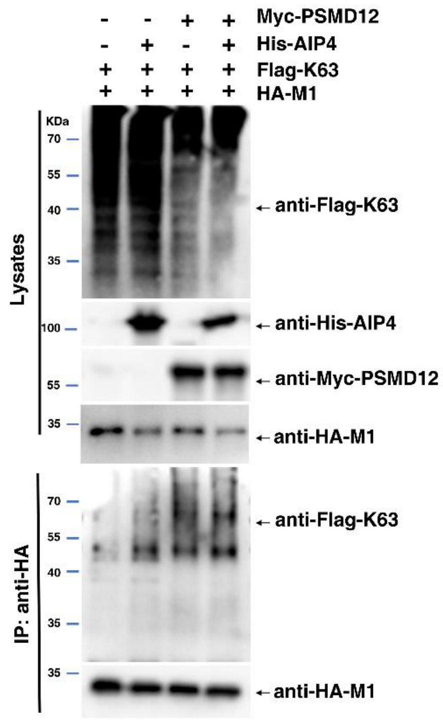

Supplementary Figure 10. AIP4 could not synergistically promote K63-linked ubiquitination of M1 with PSMD12. HEK293T cells were transfected with the indicated plasmid combinations to measure ubiquitination of M1.
